# One Patient, Three Providers: A Multidisciplinary Approach to Managing Common Neuropsychiatric Cases

**DOI:** 10.3390/jcm12175754

**Published:** 2023-09-04

**Authors:** Aynur Özge, Füsun Mayda Domaç, Nil Tekin, Esra Aydın Sünbül, Nevra Öksüz, Arife Çimen Atalar, Sümeyye Yasemin Çallı, Yağmur Sever Fidan, Ahmet Evlice, Engin Emrem Beştepe, Filiz İzci, Özge Yılmaz Küsbeci, Esra Acıman Demirel, Sibel K. Velioğlu, Mehmet Ungan

**Affiliations:** 1 Department of Neurology, School of Medicine, Mersin University, Mersin 33110, Türkiye; nvrksz@gmail.com; 2 Department of Neurology, Erenkoy Mental and Nervous Diseases Training and Research Hospital, University of Health Sciences, İstanbul 34668, Türkiye; fusundomac@yahoo.com.tr; 3 Department of Family Medicine, Izmir Faculty of Medicine, University of Health Sciences, İzmir 35330, Türkiye; niltekin33@yahoo.com; 4 Department of Family Medicine, Tepecik Education and Research Hospital, University of Health Sciences, İzmir 35330, Türkiye; 5 Department of Psychiatry, Erenköy Mental and Nervous Diseases Training and Research Hospital, University of Health Sciences, İstanbul 34668, Türkiye; dresraaydin@yahoo.com (E.A.S.); sumeyye.calli@gmail.com (S.Y.Ç.); dr.yagmurfidan@gmail.com (Y.S.F.); emremb@yahoo.com (E.E.B.); filizizci@yahoo.com (F.İ.); 6 Neurology Department, Kanuni Sultan Süleyman Education and Research Hospital, University of Health Sciences, İstanbul 34668, Türkiye; cimenatalar@gmail.com; 7 Department of Neurology, School of Medicine, Çukurova University, Adana 01330, Türkiye; aevlice@yahoo.com; 8 Neurology Department, Medical Faculty, Izmir University of Economics, Izmir 35330, Türkiye; ozgeyilmaz73@gmail.com; 9 Department of Neurology, Zonguldak Bulent Ecevit University of Medicine, Zonguldak 67100, Türkiye; esraacimandemirel@gmail.com; 10 Clinical Neurophysiology Unit, Neurology Department, Medical Faculty, Karadeniz Technical University, Trabzon 61080, Türkiye; sibelkvelioglu@hotmail.com; 11 Department of Family Medicine, Medical Faculty, Ankara University, Ankara 06100, Türkiye; mehmetungan@gmail.com

**Keywords:** neuropsychiatric cases, multidisciplinary approach, migraine, dementia, epilepsy, mood disorders, neuralgia, psychosis

## Abstract

Background: Neuropsychiatric cases require a multidisciplinary approach for effective management. This paper presented case-based discussions on migraine, dementia, epilepsy, mood disorders, neuralgia, and psychosis from the perspectives of a family physician, neurologist, and psychiatrist. The goal was to highlight the importance of collaboration between healthcare providers in managing these complex cases. Methods: The paper was based on the proceedings of the Mediterranean Neuropsychiatry Symposium, where experts from family medicine, neurology, and psychiatry came together for comprehensive case-based discussions. The CARE framework (Case Report, Appraisal, Research, and Education) was developed to guide reporting and evaluation of case reports in clinical practice. Results: Six cases were presented and discussed, highlighting the importance of a multidisciplinary approach in managing neuropsychiatric cases. The cases included chronic migraine with medication overuse, memory dysfunction with language and behavioral problems, refractory epileptic seizures with subjective sensory symptoms, bipolar affective disorder with normal pressure hydrocephalus, postherpetic neuralgia in a case with bipolar affective disorder, and psychosis with recurrent attacks with the abuse of several substances. Conclusion: A biopsychosocial multidisciplinary approach is essential for managing neuropsychiatric cases effectively on behalf of the patients and public health of the country. The CARE framework can guide the reporting and evaluation of case reports in clinical practice, ensuring that patients receive comprehensive and effective care. Healthcare providers should collaborate to provide the best possible care for patients with complex and multifaceted needs.

## 1. Introduction

Neuropsychiatric cases pose intricate challenges within clinical practice, demanding a nuanced and comprehensive approach. Conditions like migraine, dementia, epilepsy, mood disorders, neuralgia, and psychosis necessitate a biopsychosocial perspective and a collaborative, multidisciplinary strategy to ensure optimal patient management and care.

For instance, migraine, characterized by recurring headaches and associated symptoms, presents a scenario where the family physician, neurologist, and psychiatrist play distinct yet interconnected roles. While the family physician may initiate treatment, the neurologist’s diagnosis and long-term management and the psychiatrist’s support for potential mental health comorbidities create a holistic care pathway [1]. The gradual decline in cognitive function seen in dementia underscores the importance of a cohesive team effort. The family physician identifies initial signs and directs patients towards further evaluation by the neurologist and psychiatrist. Together, these professionals navigate cognitive impairments, psychological symptoms, and caregiver support, fostering a comprehensive approach to dementia care [2]. The intricate landscape of epilepsy management similarly demands the orchestration of expertise. The neurologist’s specialized knowledge takes the lead in diagnosis and treatment, with the family physician and psychiatrist supporting patients dealing with concurrent mental health conditions [3]. Mood disorders, encompassing depression and bipolar disorder, emphasize the crucial role of family physicians as the primary interface. However, a successful intervention calls for collaborative engagement with neurologists and psychiatrists. These collaborative efforts facilitate accurate diagnosis and tailored management plans [4]. Neuralgia, characterized by intense nerve-related pain, necessitates an integrated approach. While the family physician initiates treatment, the neurologist’s specialized intervention might be required for chronic cases, and the psychiatrist’s involvement could address the intertwined mental health concerns [5]. In the realm of psychosis, involving a profound disconnect from reality, the primary physician acts as the gateway. Early identification prompts referrals to both the psychiatrist for specialized assessment and the neurologist if underlying neurological factors contribute to the condition [6].

Recognizing the multifaceted nature of neuropsychiatric cases, effective management hinges upon the collaboration of family physicians, neurologists, and psychiatrists. Their collective expertise encompasses diverse facets of patient care, ensuring a holistic approach tailored to individual needs. Regrettably, the integration of such multidisciplinary approaches in routine clinical practice often faces hurdles. This manuscript bridges this gap by fostering a dynamic discussion among family physicians, neurologists, and psychiatrists. By converging distinct skill sets and insights, a comprehensive approach to patient care emerges, thus enhancing public health outcomes.

## 2. Methodology

The foundation of this paper rests upon the proceedings of the Mediterranean Neuropsychiatry Symposium. This symposium, held in Cyprus from 24th to 26th April 2023, was jointly organized by the Global Migraine and Pain Society and the Mersin Alzheimer Society. The primary objective of this symposium was to foster in-depth discussions on neuropsychiatric cases by convening experts from three distinct yet interconnected disciplines: family medicine, neurology, and psychiatry.

The process of selecting cases for discussion involved a meticulous approach. Cases were chosen to represent a spectrum of neuropsychiatric conditions, each offering unique challenges and insights. The criteria for case selection encompassed relevance, complexity, and the potential for a multidisciplinary approach to yield optimal outcomes. Central to the symposium’s outcomes was the conceptualization of a methodological acronym—CARE. The CARE framework, which stands for Case Report, Appraisal, Research, and Education (key messages), emerged during the symposium as a guide for structuring case-based statements that endorse a comprehensive, multidisciplinary approach. Each element of the CARE framework was meticulously crafted by the corresponding expert in the relevant discipline. Drawing from their own professional experiences, expert opinions, and insights from pertinent literature, these contributions aimed to capture the multifaceted aspects of each case. The synthesis of these individual contributions culminated in a comprehensive framework that captures the essence of a multidisciplinary approach in managing neuropsychiatric cases. To ensure the rigor and accuracy of the CARE framework, a rigorous review process was employed. Each author critically reviewed the collective draft, contributing their insights and expertise. This iterative process facilitated the refinement of the framework, resulting in a final version that accurately reflects the collaborative approach advocated in this paper.

In summary, the methodology for this paper revolved around the proceedings of the Mediterranean Neuropsychiatry Symposium. This symposium served as a platform for experts from family medicine, neurology, and psychiatry to collaboratively discuss and synthesize their insights into a coherent framework—CARE. Through the concerted efforts of these experts, the CARE framework embodies the essence of a multidisciplinary approach in managing complex neuropsychiatric cases.

## 3. Case 1: Chronic Migraine with Medication Overuse

### 3.1. History of Cases by N.Ö.

A 28-year-old woman worked as a lawyer and presented to our clinic with a complaint of persistent headaches since her teenage years. While she used to experience 7–8 attacks per month, she had been having headaches almost every day for the past 2 years. She described the headaches as occasional pressing sensations, often throbbing, accompanied by symptoms of sensitivity to light (photophobia), sensitivity to sound (phonophobia), nausea, vomiting, sensitivity to smell (osmophobia), and dizziness. Her headaches were usually severe, with a visual analog scale score (VAS) of 8–9. During some attacks, she experienced cranial autonomic symptoms such as redness and tearing of the eyes. She also experienced symptoms of allodynia. She did not have any aura preceding the headaches and found relief by lying down in a dark room and taking ibuprofen at the onset of an attack. She reported that her headaches worsened during her menstrual cycle. The attacks typically lasted for about 12–24 h. In her medical history, she had been diagnosed with essential tremor and irritable bowel syndrome. It was notable that she experienced motion sickness during her childhood and had undergone surgery for recurrent ear infections at the age of 13. Her family history revealed that her sister and mother were also diagnosed with migraine. Her father had essential tremor. Her neurological examination was normal, and her body mass index was 24.36 kg/m^2^. A cerebral magnetic resonance imaging (MRI) taken approximately 4 years ago showed no abnormalities. She also had comorbid psychiatric symptoms, including anxiety and relationship issues with her parents. Her Migraine Disability Assessment (MIDAS) score was 50, indicating significant disability, and her Headache Impact Test-6 (HIT-6) score was 61. At the time of her visit, she was not using any prophylactic medications but was using painkillers almost daily. She had previously used fluoxetine and propranolol at the appropriate dose and duration but had no benefit. She was diagnosed with chronic migraine (CM) and medication overuse headache (MOH). Treatment for the patient included topiramate as a prophylactic medication and recurrent greater occipital nerve (GON) blockades as a bridging treatment. Regular exercise was also recommended. Although she initially refused to see a psychiatrist despite repeated recommendations, it was strongly advised again when her progress with treatment alone was not sufficient. Eventually, she agreed to be evaluated by a psychiatrist and experienced significant improvement with the additional treatment.

### 3.2. Neurological Point of View by F.M.D.

#### 3.2.1. Comprehension

The case discusses a 28-year-old female lawyer who has been suffering from persistent headaches since adolescence, which have developed into chronic migraines with frequent attacks. Her symptoms include throbbing headaches with sensitivity to light, sound, smell, and nausea. She also experiences cranial autonomic symptoms, allodynia, and exacerbations during her menstrual cycle. A family history of migraines further supports her diagnosis.

#### 3.2.2. Appraisal

Migraine, a globally prevalent chronic ailment, extends beyond mere headaches, exerting substantial impacts on social, academic, and work aspects, hampering productivity [7]. Its burdens are evident during both attack and interictal periods [8]. The disease’s economic repercussions, whether direct or indirect, are substantial [9]. The transition from episodic to chronic migraine is influenced by behavioral, social, genetic, and environmental factors [10]. Elevated attack frequency, medication overuse, inadequate treatment, and concurrent comorbidities contribute to this shift [1]. Triggers like stress, menstrual cycle, and lifestyle factors can precipitate and exacerbate attacks, with gender-related effects on susceptibility and severity [11]. Migraine comorbidities span vascular, neurological, sleep-related, gastrointestinal, allergy-related, pain-related, and psychiatric domains [8]. Medication overuse’s role in the pain matrix is noteworthy, resulting in chronic medication overuse headache [12]. This patient, embodying psychiatric and gastrointestinal comorbidities, experiences sex-linked menstrual cycle exacerbations. A familial migraine history and escalating attack frequency signify migraine’s chronicity. Additionally, medication overuse headache arises due to frequent analgesic use. Limited response to effective prophylactics suggests possible resistant migraine classification [13]. Considering this profile, calcitonin gene-related peptide (CGRP) antagonists offer a suitable treatment avenue. Non-pharmacological strategies, encompassing lifestyle modifications, trigger management, and cognitive behavioral therapy, should be initial steps for intervention [1]. Furthermore, understanding the gender-based influence on clinical presentation and progression is paramount in personalized migraine management.

#### 3.2.3. Research

The effects on quality of life, migraine-associated disability, ictal and interictal migraine-related burden, triggers for migraine attacks, and comorbidities influencing the disease course should be taken into consideration during migraine management [14].

The first step in reducing headache attacks is to avoid/desensitize triggers. If preventive therapy is required, it should be determined on a case-by-case basis, considering comorbidities, to choose an effective agent. Nonpharmacological therapies, such as neuromodulatory devices, acupuncture, and cognitive behavioral therapy, can be used as adjunctive or standalone approaches for both acute and prophylactic treatment. First-line pharmacological agents for migraine prevention include beta-blockers (propranolol, metoprolol, atenolol), angiotensin-converting enzyme inhibitors (candesartan), and antiepileptics (topiramate), while tricyclic antidepressants (amitriptyline), calcium antagonists (flunarizine), and antiepileptics (sodium valproate) are considered second-line medications. Botulinum toxin and CGRP antagonists are third-line options [1]. CGRP, known for its role in pain transmission within the trigeminovascular complex and neurogenic inflammation, is a target in migraine treatment. CGRP antagonists (erenumab, fremanezumab, galcanezumab) have shown long-term effectiveness and safety, making them suitable for prophylactic treatment of both episodic and chronic migraine [15].

Peripheral nerve blockade, primarily targeting the greater occipital nerve, is widely used, effective, and well tolerated in both acute and preventive migraine management [16]. However, as there is no consensus yet regarding the technique, dosage, and application interval, further prospective studies are needed. After initiating or altering prophylactic agents, treatment response (pain relief, adverse effects, complications) should be assessed within 2–3 months to determine treatment success [1]. Medication overuse should be ruled out and, if present, treated effectively. Patient education on the association between headache and overused acute medications is crucial, followed by immediate withdrawal or tapering of the overused agent and initiation of preventive therapy. Topiramate (up to 200 mg), Onabotulinumtoxin-A, and CGRP antagonists have demonstrated effectiveness in prevention. During withdrawal, amitriptyline, corticosteroids (prednisolone/prednisone), and antiemetics can alleviate symptoms. Regular follow-up is necessary due to the risk of relapse after withdrawal therapy [17,18].

#### 3.2.4. Agreement

The case and our discussion underscore the significant impact of migraines, extending beyond mere headaches, affecting various aspects of life including social, academic, and work-related domains. The recognition of both the economic consequences and the transition from episodic to chronic migraine influenced by behavioral, genetic, and environmental factors aligns with current understanding. The emphasis on triggers like stress and lifestyle factors contributing to attack occurrence resonates with existing knowledge. Moreover, the acknowledgment of migraine comorbidities spanning multiple domains and the relevance of medication overuse in pain management is well-founded.

#### 3.2.5. Disagreement

However, the characterization of CGRP antagonists as third-line options might not fully reflect their growing prominence as effective early-line prophylactic treatments for migraines. Current research indicates their suitability for both episodic and chronic migraine due to their established long-term effectiveness and safety. The discussion on peripheral nerve blockade’s wide usage and effectiveness is agreed upon, but the uncertainty around technique, dosage, and application interval calls for additional standardized research to refine its application.

#### 3.2.6. Reflection

Overall, the case study highlights the intricate nature of migraines and the need for a comprehensive approach. The importance of considering various factors such as triggers, comorbidities, and tailored treatments is well-stated. The emphasis on patient education and a multidisciplinary approach aligns with the holistic understanding of migraine management.

In conclusion, the case provides a comprehensive view of migraine’s impact and management. While there are points of agreement and disagreement, the patient’s well-being and improved quality of life remain the central focus.

#### 3.2.7. Education (Key Messages)

- Migraine often presents with comorbid conditions and is among the most common psychiatric disorders;
- Careful evaluation of accompanying symptoms and tailored management plans are essential;
- A multidisciplinary approach is crucial, particularly for chronic migraine patients. Attention should be given to the social stigmatization concerns of patients;
- Primary care physicians play a pivotal role as the first point of contact for patients with new-onset headaches, and they should be vigilant about medication overuse. If medication overuse is present, it should be effectively treated by a neurologist to enhance the success of migraine therapy.

### 3.3. Psychiatric Point of View by E.A.S.

#### 3.3.1. Comprehension

The case examines migraines from a psychiatric perspective, highlighting stress as a prominent trigger and exploring the connection between migraines and psychiatric comorbidities. A case study of a 28-year-old lawyer with chronic migraines was presented, along with discussions on stressors, psychosomatic aspects, and the biopsychosocial model’s relevance in understanding migraines.

#### 3.3.2. Appraisal

Stress triggers migraines in around 70% of individuals, particularly those with chronic daily migraine [19]. Psychological stressors, like trauma and social stress, contribute significantly to the overall migraine burden. In the case of a stressed 28-year-old lawyer, her parents’ aging adds to her stressors. Further assessment requires a comprehensive psychiatric interview and mental state examination. Psychosomatic disorder, marked by unexplained physical symptoms, often involves excessive concern about symptoms, impairing functionality [20]. Migraines show psychosomatic tendencies, often coexisting with other similar conditions. For instance, this patient has a history of irritable bowel disease. People with psychosomatic disorders tend to attribute issues to medical causes, avoiding acknowledging psychological distress. Similarly, this patient resisted psychiatric help despite doctor recommendations. Numerous studies affirm the high prevalence of psychiatric comorbidities like depression, anxiety, and post-traumatic stress disorder in migraine sufferers. Left untreated, these conditions escalate chronicity and disability while degrading quality of life and treatment outcomes. In this case, anxiety symptoms are present, demanding further investigation into their severity. The biopsychosocial model highlights the interplay of biological, psychological, and social factors in chronic headaches such as migraines. Psychological interventions like cognitive behavioral therapy (CBT), relaxation techniques, and biofeedback hold significance [21].

#### 3.3.3. Research

Migraine, one of the most prevalent disorders globally, is characterized by high levels of psychological comorbidity [22]. As a result, it is viewed as a biopsychosocial condition [23], influenced by cognitive, emotional, environmental, and biological factors. Among those who consult their family doctor for headaches, 28% exhibit clinically significant levels of anxiety or depression [24]. Psychiatric symptoms accompanying migraines have significant consequences, as psychiatric conditions are risk factors for the transformation of migraines into a chronic form [25]. Additionally, individuals with migraines and comorbid psychiatric disorders utilize more healthcare resources than migraineurs without such conditions. Recognizing this comorbidity enables improved patient management through targeted first-line treatments addressing both conditions. The biopsychosocial model of migraines suggests the utilization of psychological treatment options, such as cognitive behavioral therapy (CBT), relaxation techniques, and biofeedback, enabling patients to develop strategies for prevention and acute management. Reviews of randomized controlled trials assessing the effectiveness of behavioral treatments have demonstrated a 32% reduction in headache frequency compared to 5% in control groups [26].

In conclusion, the comorbidity between migraines and psychiatric disorders has been extensively investigated, yet the underlying mechanisms remain poorly understood. Given the significant impact of psychiatric comorbidity on migraine evolution, treatment strategies, and overall outcomes, further research on this topic is crucial.

#### 3.3.4. Agreement

The recognition of stress as a significant trigger for migraines and its impact on individuals, especially those with chronic daily migraines, is in line with established knowledge. The understanding of migraines as a biopsychosocial condition influenced by multiple factors resonates with current understanding. The acknowledgment of the prevalence of psychiatric comorbidities, such as depression, anxiety, and post-traumatic stress disorder, in individuals with migraines is well-supported. The emphasis on the importance of considering psychological treatment options, like cognitive behavioral therapy (CBT), relaxation techniques, and biofeedback, aligns with evidence-based approaches to managing migraines.

#### 3.3.5. Disagreement

However, while stress is indeed a common trigger for migraines, the statement that it affects nearly 70% of individuals, especially those with chronic daily migraines, might need further clarification. The precise percentage can vary based on different studies and populations. The mention of a 32% reduction in headache frequency through behavioral treatments in comparison to 5% in control groups is insightful. Still, it is important to note that the effectiveness of such treatments can depend on various factors and might differ between individuals.

#### 3.3.6. Reflection

The case study underscores the need for a comprehensive understanding of migraines, considering both biological and psychological aspects. The emphasis on recognizing the impact of psychiatric comorbidities on migraine evolution and management reflects a patient-centered approach.

#### 3.3.7. Education (Key Messages)

- Investigations into migraine comorbidity have established its association with various psychiatric conditions, highlighting the importance of referral to psychiatry for patients;
- Adopting a biopsychosocial model is crucial in understanding headaches, and psychological treatment options, such as CBT, relaxation techniques, and biofeedback, should be considered;
- Stress is a common trigger for migraines, affecting nearly 70% of individuals, especially those with chronic daily migraine. Psychological stressors, including trauma and social stress, contribute to the overall burden of the disease;
- Psychiatric comorbidities, including depression, anxiety, and post-traumatic stress disorder, are prevalent in individuals with migraines. If left untreated, these conditions can increase migraine chronicity, disability, and diminish quality of life;
- Migraine is a biopsychosocial condition influenced by biological, psychological, and social factors. Cognitive behavioral therapy (CBT), relaxation techniques, and biofeedback are important psychological treatment options to consider.

### 3.4. Family Physician Point of View by M.U.

#### 3.4.1. Comprehension

In Family Practice/General Practice (FP/GP), embracing a biopsychosocial approach to comprehending the evolving nature of headaches over time holds paramount importance. The ability to care for patients longitudinally, access their medical history, and track health patterns positions FP/GPs to potentially expedite diagnoses. However, challenges arise when dealing with chronic headaches extending back to 2017, necessitating a comprehensive data-driven approach.

#### 3.4.2. Appraisal

The Longitudinal and Holistic Approach to Headache Cases: Emphasizing the Biopsychosocial Perspective. In Family Practice/General Practice (FP/GP), a biopsychosocial approach to understanding the effects of time on headache diagnosis plays a vital role. When a FP/GP cares for the same patient over an extended period, with access to various medical records, it becomes a reliable source for observing changes in health patterns, potentially leading to earlier diagnoses. However, for a patient experiencing a specific type of headache since 2017, gathering more data becomes essential to ensure a safe and accurate diagnosis.

Managing Expectations: The Challenge of Matching Patient and Physician Perspectives. Patients often expect a quick, magical solution to their health problems. However, in our daily practice, we frequently encounter patients who avoid referrals due to fears of stigma or issues with the healthcare system. Simultaneously, doctors may feel hesitant to refer patients, preferring to continue with their current approach to avoid potential risks. In such cases, it becomes the responsibility of the FP/GP to provide relevant data supporting the need for referrals. Patients, too, should take charge of their healthcare by making their personal health records accessible to the healthcare professionals.

The Significance of Headache Diaries: Considering that psychiatrists have an average consultation length of 15 to 40 min and FDs/GPs have only 7 to 15 min, it becomes challenging to adopt a comprehensive biopsychosocial approach during these short interactions. To effectively implement this approach, it is crucial to establish long-term follow-ups and build professional relationships with patients, wherein patients also take an active role in their care. Therapeutic Patient Education (TPE) is an essential tool in this context. TPE empowers individuals with chronic diseases to acquire and maintain skills for optimal disease management. Updating and revising resources like the WHO’s guide on TPE can assist Member States in strengthening their TPE programs. It is hoped that a new guide will support Member States to reinvigorate their approach to TPE and move forward with their programs. The main guide was supported by a series of country case stories/boxes of good practice as illustration, and a collation of relevant tools. That is what we did also here in N. Cyprus.

Patient Empowerment as a Solution for Non-Communicable Diseases (NCDs): NCDs account for 70% of global all-cause mortality, making patient empowerment a sustainable proposal for addressing such conditions. The paradigm shift towards an empowerment philosophy in chronic disease care is influenced by social changes promoting principles like “self-determination” and “autonomy”. Patients shall be empowered and the communication shall be at the same level by both sides. That is why we need tools like patient headache diary which makes this relation visible and creates a ground to discuss together objectively not depending on only perceptions or prejudgment. Disease management and education programs based on empowerment have shown promising results in improving disease outcomes and encouraging shared decision-making between patients and healthcare professionals.

Considering Past Medical History in Diagnosis: When diagnosing headaches, it is essential to consider a patient’s past medical history, including conditions such as atopic rhinosinusitis and metabolic disorders, which may be interconnected with migraines. A thorough review of medical records can unveil potential patterns or associations between chronic headaches and other repetitive conditions, leading to earlier detection of rare diagnoses like NENs (Neuroendocrine Neoplasms). FP/GPs play a critical role in being vigilant about chronic conditions and considering relevant possibilities to ensure timely and accurate diagnoses.

Atopic rhinosinusitis and metabolic disorders as a mismatching cloudy part of migraine diagnosis. By this part, we see that we need to think about the past/history of a person in our records. If there is a history like several ear operations, or GI symptoms, allergies, like repetitive conditions, they may be associated with a chronic headache later.

Some rare diagnoses like NENs are always dependent on a FP/GP to think on when seeing a patient “coming back with the same problems”, like GI symptoms or headache. If we do not consider it, there is a high likelihood that nobody else will, given that 70 percent of NEN cases are diagnosed late. The same applies to the chronic migraine pain. Death is a fear possibly for all of us, but having a life full of pain may be devastating for many people, and living in good quality, without pain, is possible if we suspect the symptoms early, as always.

What is to say for this case as a GP/FD? I can simply say that this is a headache for us. And again, in such an intense and broad work, we would like to know when and where and how to refer to put all on the safe side.

In conclusion, addressing headaches from a longitudinal and holistic perspective requires effective patient–physician communication and collaboration. By embracing the biopsychosocial approach, incorporating headache diaries, promoting patient empowerment, and considering past medical history, we can enhance the accuracy and safety of headache diagnoses. Ensuring appropriate referrals and timely interventions will lead to better patient outcomes, reducing the burden of pain and improving overall quality of life.

#### 3.4.3. Research

A comprehensive understanding of chronic migraine (CM) and its various aspects is crucial for effective management. In this session, we aimed to delve into the research surrounding CM, exploring its clinical profile, comorbid psychiatric symptoms, genetic and environmental factors, impact of medication overuse, efficacy of prophylactic treatments, effectiveness of nerve blockades, and the prevalence of psychiatric comorbidity. From a general practitioner’s perspective, CM presents a complex and challenging condition to diagnose and manage. Understanding the clinical profile of CM helps GPs recognize the specific features that differentiate it from episodic migraines and other headache disorders, enabling more accurate diagnoses and tailored treatment plans.

By analyzing case studies and conducting cross-sectional and longitudinal studies, researchers have shed light on the unique characteristics and challenges associated with CM [27]. Additionally, investigations have focused on the association between CM and psychiatric symptoms, highlighting the need for comprehensive assessment and management [28]. The association between CM and psychiatric symptoms is an essential consideration for GPs. Many patients with chronic migraines also suffer from anxiety, depression, or other psychiatric conditions, which can further complicate treatment strategies. A comprehensive assessment that addresses both the physical and psychological aspects of CM is vital for a holistic approach to patient care. Genetic and environmental factors have also been explored, revealing their contribution to CM development and progression [29]. Understanding these influences can guide GPs in recommending lifestyle modifications and identifying potential genetic predispositions. Furthermore, the impact of medication overuse on CM treatment outcomes has been investigated, emphasizing the importance of addressing this issue [30]. GPs play a critical role in educating patients about appropriate medication use and implementing strategies to prevent medication overuse and its associated complications.

Prophylactic treatments like topiramate have been evaluated for their efficacy and safety in managing CM. However, careful monitoring of patients’ response to treatment and potential side effects is necessary to optimize the benefits and minimize adverse reactions.

Additionally, the effectiveness of nerve blockades, particularly greater occipital nerve blockades, as a bridging treatment has been assessed [1,31]. GPs should be aware of availability and effectiveness of nerve blockades as an option.

Finally, considering the high prevalence of psychiatric comorbidity in CM, GPs should adopt an integrated approach, working in tandem with mental health professionals to address both the physical and emotional aspects of the condition. This collaborative effort ensures a comprehensive and personalized treatment plan, which is essential for managing CM effectively. By thoroughly examining these research areas from a general practitioner’s viewpoint, and with the specialist experiences, we aim to enhance our understanding of CM and facilitate the development of more effective management strategies in primary care settings.

#### 3.4.4. Agreement

The research aligns with the consensus that comprehensive assessment of CM patients is essential. Acknowledging the association between CM and psychiatric symptoms is vital for delivering holistic care. Moreover, understanding genetic and environmental influences in CM underscores the need for tailored treatment strategies. The research confirms that a collaborative approach between patients and healthcare professionals is pivotal for effective CM management.

#### 3.4.5. Disagreement

While the research highlights the importance of comprehensive patient assessment, the reality is that brief consultation times present a challenge. Practically, it is complex to incorporate a holistic biopsychosocial approach within these constrained interactions. Therefore, the ideal of comprehensive assessment might face limitations due to time constraints in actual clinical settings.

#### 3.4.6. Reflection

The extensive research and the practical insights gained lead us to reflect on the ongoing efforts to enhance CM management. The need for an integrated approach that addresses both the physical and psychological aspects of CM become evident. This journey is marked by the continuous pursuit of refining our understanding and methodologies to provide the best possible care.

#### 3.4.7. Education (Key Messages)

- Chronic migraine (CM) is a complex condition that requires a comprehensive understanding of its clinical profile, including symptom patterns, triggers, and associated comorbidities;
- Recognizing the association between CM and psychiatric symptoms is crucial for holistic management in primary care and in tertiary level, as addressing mental health concerns can significantly impact treatment outcomes and overall quality of life;
- Genetic and environmental factors play a role in the development and progression of CM, highlighting the importance of considering individual susceptibility and potential triggers;
- Medication overuse can worsen CM symptoms and hinder treatment effectiveness. Patients should be educated about the risks of overusing painkillers and the importance of adhering to appropriate medication guidelines;
- Integrated treatment approaches, such as combining prophylactic medications, nerve blockades, lifestyle modifications (including regular exercise), and psychological interventions, offer a comprehensive and tailored approach to managing CM and improving patient outcomes.

## 4. Case 2: Memory Dysfunction with Language and Behavioral Problems

### 4.1. Summary of Cases by A.E.

A 71-year-old right-handed female with 13 years of education (key messages) presented with a 6-year history of memory loss, social withdrawal, and strange behaviors since her youth. It was reported that she was dependent on assistance for activities of daily living, including homework and grocery shopping. Her medical history included hypertension, while her family history did not show any significant association with dementia. Due to the patient’s lack of cooperation and unresponsiveness to questions, a comprehensive neuropsychological evaluation could not be conducted. The neurological examination revealed a depressive mood. She was prescribed Donepezil 10 mg/day, Memantine 20 mg/day, Duloxetine 30 mg/day, Perindopril 5 mg/day, and Risperidone 0.5 mg/day. The routine biochemistry and hemogram results were within normal limits. The patient’s cranial MRI demonstrated cortical atrophy.

### 4.2. Neurological Point of View by Ö.Y.K.

#### 4.2.1. Comprehension

We examined the case of a 71-year-old right-handed woman with 13 years of education, who presented with a 6-year history of memory loss, social withdrawal, and unusual behaviors. Dependent on daily living assistance, her medical history included hypertension. Although her family lacked a significant dementia association, her unresponsiveness thwarted a comprehensive neuropsychological evaluation. She received a regimen involving Donepezil, Memantine, Duloxetine, Perindopril, and Risperidone. Routine biochemistry and hemogram results were normal, and cranial MRI showed cortical atrophy.

#### 4.2.2. Appraisal

The patient, who has exhibited peculiar behaviors since youth, has been experiencing increased complaints of forgetfulness and social withdrawal. However, due to the patient’s lack of cooperation and unresponsiveness to questions, a comprehensive neuropsychological evaluation could not be conducted. The patient’s cranial MRI revealed cortical atrophy, and they had previously received a diagnosis of dementia and depression at the center where they were previously treated, leading to the initiation of treatment.

In the geriatric population, there is often an overlap between dementia and depression, and individuals with depression may be mistakenly diagnosed with dementia. Depression is the most prevalent mood disorder among older adults, with a prevalence rate of up to 6.7% in individuals aged 60 and above. This rate increases with age, reaching up to 85% in individuals in their 80s [32]. Another challenge in diagnosing depression is the potential confusion between depression symptoms and dementia symptoms, particularly in the elderly. Late-onset depression and early-stage dementia symptoms can sometimes exhibit overlapping characteristics, making it difficult to differentiate between the two. Additionally, depression is the most common neuropsychiatric symptom accompanying Alzheimer’s disease [33].

Careful observation of semiological clues in the outpatient setting can provide valuable insights into the patient’s condition. A thorough assessment of mood changes, cognitive abilities, and functional impairment, along with input from family members or caregivers, contributes to a more accurate diagnosis. One of the most crucial factors in distinguishing between depression and dementia is the onset of symptoms. In dementia, symptoms tend to have a slower onset, while depression symptoms typically manifest more rapidly [34]. Another important clue is that patients with depression often complain more about cognitive impairments, whereas individuals with dementia frequently deny any memory complaints and state that they sought medical attention because of their family’s concerns [35]. In this case, the patient’s family could not provide precise information about the onset of the patient’s symptoms, but the patient did not have significant complaints, and it was their family who brought them to the hospital.

When both depression and dementia symptoms coexist, neuropsychological tests are crucial. However, in the case of the patient described, tests could not be conducted due to the patient’s lack of cooperation and unresponsiveness to questions. It is important to note that dementia does not solely manifest as memory impairment; it can also involve behavioral and language disturbances, as seen in frontotemporal dementia [36]. Therefore, in the evaluation of patients, it is imperative to prioritize obtaining a detailed medical history from the patient and their family, conducting comprehensive neuropsychological assessments, and adopting a multidisciplinary approach that involves collaboration with the psychiatry clinic. This approach is essential for achieving an accurate diagnosis and implementing effective patient management and follow-up.

#### 4.2.3. Research

Neuropsychiatric symptoms are prevalent among patients with Alzheimer’s disease (AD), posing challenges in their identification and management. This difficulty stems from the intricate interplay between symptoms that may be attributed to psychiatric disorders and those directly arising from dementia pathology. Psychiatric symptoms such as depression, anxiety, agitation, psychosis, and sleep disturbances frequently coexist with cognitive impairments in individuals with AD. However, the underlying mechanisms linking these symptoms to the neurodegenerative processes of AD remain elusive. Aalten and colleagues reported findings from the largest European database, the European Alzheimer’s Disease Consortium (EADC), which identified four distinct sub-syndromes of Neuropsychiatric Symptoms (NPS). These sub-syndromes include hyperactivity (aggression, disinhibition, irritability, aberrant motor behavior, and euphoria), psychosis (delusions, hallucinations, and sleep disorders), affective symptoms (depression and anxiety), and apathy (apathy and appetite disorders) [37]. Over 80% of individuals with dementia, including those with cognitive impairment associated with Alzheimer’s disease (AD), exhibit at least one neuropsychiatric symptom since the onset of cognitive decline [38]. However, despite their high prevalence, neuropsychiatric symptoms in AD patients are often under-recognized and inadequately managed [39]. Addressing these symptoms requires comprehensive assessments, including detailed clinical evaluations, neuroimaging techniques, and standardized rating scales for symptom severity. A multidisciplinary approach involving collaboration among neurologists, psychiatrists, geriatricians, neuropsychologists, and other healthcare professionals is crucial for comprehensive management. Comprehensive assessment, multidisciplinary collaboration, targeted non-pharmacological interventions, and appropriate use of pharmacotherapy are crucial elements for effectively addressing and managing neuropsychiatric symptoms in individuals with AD.

#### 4.2.4. Agreement

The research resonates with the importance of comprehensive assessments and interdisciplinary collaboration. Neuropsychiatric symptoms are prevalent in AD patients but often overlooked. Addressing them mandates a collective approach encompassing various specialties. Early recognition and personalized care remain pivotal in managing these symptoms.

#### 4.2.5. Disagreement

While the research accentuates comprehensive assessment and collaboration, the reality is complicated by uncooperative patients. Conducting neuropsychological tests in such cases poses a challenge. The ideal approach may face practical constraints due to patient cooperation issues.

#### 4.2.6. Reflection

Reflecting on the research and the case, it is clear that improved management of neuropsychiatric symptoms in AD necessitates a thorough interdisciplinary approach. The amalgamation of insights from research and practical experience guides us towards better patient outcomes.

#### 4.2.7. Education (Key Messages)

- Neuropsychiatric symptoms are highly prevalent in individuals with AD, affecting over 80% of patients since the onset of cognitive decline;
- Despite their high prevalence, neuropsychiatric symptoms in AD are often under-recognized and inadequately managed;
- Managing neuropsychiatric symptoms in AD requires a multidisciplinary approach involving collaboration among neurologists, psychiatrists, geriatricians, neuropsychologists, and other healthcare professionals;
- Early recognition and comprehensive assessment of neuropsychiatric symptoms in AD are essential for timely intervention;
- Providing person-centered care is crucial when managing neuropsychiatric symptoms in AD.

### 4.3. Family Physician Point of View by N.T. and M.U.

#### 4.3.1. Comprehension

In this exploration, we delved into the viewpoints of family physicians, regarding the nuanced realm of dementia management. Their insights provide a comprehensive understanding of the challenges and strategies adopted in diagnosing, caring for, and supporting dementia patients.

#### 4.3.2. Appraisal

Family physicians serve as the initial point of contact in healthcare services. They have a crucial role in the timely diagnosis of dementia, staging the disease, communicating the diagnosis to patients and their families, and managing patients after diagnosis [40]. It is well known that the incidence of chronic diseases rises with age, and dementia and multimorbidity lead to health deterioration and functional decline [41]. The primary goal is to preserve functionality when managing elderly patients, who are increasingly seeking care in family medicine. Family physicians adopt a holistic approach in evaluating individuals, regardless of age, gender, or specific organ/system concerns. Consequently, they possess experience in addressing multiple concurrent issues and conducting continuous monitoring [40].

Dementia patient follow-up and care benefit from a collaborative approach involving family and community support. Family physicians not only provide solutions to patients but also address the problems faced by caregivers throughout all stages of dementia, including advanced care planning. Furthermore, family physicians serve as a valuable source of information for patients’ relatives and caregivers. They can refer patients, families, and caregivers to community service organizations like the Alzheimer’s Association, which offer information, support, and education (key messages) regarding dementia [40,42].

#### 4.3.3. Research

Dementia patients have extensive needs that extend beyond the boundaries of traditional medical practice, encompassing both pharmacological and non-pharmacological interventions. Future research should focus on social policy initiatives that promote preventive lifestyle behaviors and the development of healthcare programs to support the growing number of dementia patients. The significance of primary healthcare in this domain is evident [43]. It is essential to enhance these services and engage in multidisciplinary studies involving related disciplines such as neurology and psychiatry.

#### 4.3.4. Agreement

The insights from us align with the broader consensus on the importance of family physicians in dementia care. Their emphasis on collaboration, holistic care, and multidisciplinary approaches echoes the broader healthcare narrative.

#### 4.3.5. Disagreement

While the perspectives highlight the significance of primary healthcare, practical challenges may arise in terms of resource allocation, interdisciplinary coordination, and the integration of social policy initiatives.

#### 4.3.6. Reflection

Our perspectives offer a roadmap for improved dementia care. They underscore the complexity of the role while acknowledging the need for innovative solutions to address the multifaceted challenges of dementia management.

#### 4.3.7. Education (Key Messages)

- The role of family physicians in the early diagnosis, timely referral, and effective treatment of dementia is increasingly important in daily practice;
- Family physicians should take on the management process of patients, particularly in collaboration with neurology, psychiatry, and geriatric specialists;
- Strategies should be developed to safeguard functionality and quality of life in managing not only dementia but also other comorbid conditions that may arise in elderly patients;
- Family physicians should be knowledgeable about resolving family and caregiver issues and accessing social support resources (e.g., Alzheimer’s associations) when caring for dementia patients;
- To combat polypharmacy in dementia patients, family physicians should prioritize rational drug use and effectively implement quaternary prevention measures by staying well-informed about the topic.

## 5. Case 3: Refractory Epileptic Seizures with Subjective Sensory Symptoms

### 5.1. Summary of Cases by A.Ç.A.

A 23-year-old right-handed male presented with refractory seizures since he was 7 years old. In his medical history, he had extended postnatal jaundice after birth and febrile convulsion when he was 3 years old. He did not have any parental consanguinity or any siblings with epilepsy. His systemic and neurological examinations were normal except for a slight cognitive performance deficit. He presented with two major types of seizures: focal aware seizures (up to 10 seizures/day) and bilateral tonic-clonic seizures with impaired awareness (up to 8–20 seizures/month). Focal aware seizures began with tingling at the back of his head and followed by visual auras with a duration of 30–60 s presenting as blindness or seeing geometrical shapes such as squares at the right visual area. These auras did not exist at the beginning of his seizures and were added to the focal seizures in the last 2 years. The propagation of focal seizures was presented as deviation of both eyes to the right and up, swallowing followed by oral and manual automatisms. He had been wandering around and feeling micturition at the postictal phase. He had only 2 years of seizure-free period between 9 and 11 years of age without any other remission. He had comorbid depression and anxiety disorder diagnosed by psychiatric evaluation. He currently is using Carbamazepine 1200 mg/day, Lacosamide 400 mg/day, Levetiracetam 3000 mg/day and has been using a Vagal Nerve Stimulation (VNS) device for 5 years. The interictal and ictal video electroencephalography (EEG) recordings showed right temporo-occipital spike and waves. MRI showed bilateral occipital hyperintensities dominant on the right hemisphere. His neuropsychological tests revealed complex attention, visual spatial perception, executive and visual memory deficits along with verbal memory and visual naming disorders showing bilateral but right hemisphere dominant occipito-frontal involvement. These neurophysiological findings might point to the right occipital region as the source of his seizures. Positron emission tomography (PET) could not localize any specific source for seizures. We proposed long term video-EEG monitoring (VEM) with invasive electrodes and informed the patient about an epilepsy surgery option (probably right occipital region) to the patient but he did not accept an invasive investigation or a surgery.

### 5.2. Neurological Point of View by S.K.V.

#### 5.2.1. Comprehension

Within this context, we examined a case study that delved into the complexities of refractory epileptic seizures with subjective sensory symptoms. A.Ç.A.’s evaluation provides a comprehensive understanding of the patient’s medical history, symptoms, and treatment journey.

#### 5.2.2. Appraisal

The patient has refractory focal and generalized tonic-clonic seizures. Although his EEG and neuropsychological evaluation results show the same localization, a definite seizure localization is not possible. MRI showed bilateral occipital lesions and seizure semiology did not support the right hemisphere seizure onset. It was necessary to apply an invasive procedure to detect the seizure onset area.

In patients with refractory seizures—like this patient—it is mandatory to differentiate a real epileptic seizure from psychogenic attacks. Subjective symptomatology of non-motor focal seizures—visual symptoms like those of our patient—is always a problematic issue. VEM is accepted as the gold standard for differentiating non-epileptic from epileptic seizures but it might not be available in all neurology centers [44]. Therefore, semiological clues are extremely valuable in differential diagnosis. A fluctuating course, ictal eye closure, crying, duration exceeding two minutes, non-stertorous postictal breathing pattern, lack of amnesia or postictal state, and several motor signs were found to be most frequent characteristics of psychogenic seizures [45,46,47]. Our patient had real epileptic seizures recorded on video-EEG with the etiology without any clues related to psychogenic seizure semiology.

Visual auras can be a part of a focal seizure or a migraine attack [48]. It might sometimes be challenging to differentiate auras between these two disorders but there are some certain differences that need to be questioned when evaluating the patient. Epileptic auras tend to be shorter (seconds to minutes) with a persistent lateralization, more frequent than migraine auras, colored hallucinations are more common and postictal headache sometimes might follow the epileptic seizures [48]. The visual auras of the patient had a short duration of 30–60 s, presenting as blindness or seeing geometrical shapes, always recurring at the right visual area, and were followed by oculomotor manifestations supporting the features of an epileptic seizure.

The management of this patient was very compelling since he had frequent multidrug-resistant refractory seizures. Refractory epilepsy management requires a tailored treatment approach with properly chosen antiseizure drugs in a good combination at adequate doses [49] Although the patient had proper anti-seizure medical management and a VNS device to control his seizures, he still had refractory focal and generalized seizures and related cognitive deficits. The most appropriate approach to this patient would be a multidisciplinary approach with a team including a psychiatrist, a family physician, and an epileptologist to rearrange the medical treatment plan and a close follow-up of the seizures of the patient. Epilepsy surgery is another treatment option for this patient in order to decrease seizure frequency but the patient did not accept epilepsy surgery. Patients with drug-resistant epilepsy have an increased rate of psychiatric disorders like depression, anxiety disorders, and, to a lesser extent, bipolar disorder and other forms of psychosis [50]. Our patient had depression and anxiety that required psychiatric treatment, which is an important step for good seizure control.

#### 5.2.3. Research

Since the etiology of many brain disorders are multifactorial, it is obvious that there is a need for multidimensional, namely neuro-biopsychosocial, approach when evaluating a patient with a neurological disorder [51]. Especially for patient groups with epileptic disorders, movement disorders, multiple sclerosis, headache, and chronic pain, a multidisciplinary vision should be integrated into the treatment plans of these patients. When we look from the epilepsy perspective, patients with new onset seizures usually apply to primary care physicians (PCP) as the first step and the basic care and follow-ups of these patients are provided. Physicians who are less comfortable with disease entities or who want to learn more about disease usually refer their patients to a neurology specialist particularly to determine or confirm the diagnosis and appropriate treatment [52,53].

Another discipline that is as important as primary care is psychiatry. Neurology and psychiatry are two disciplines that come from common historical origins but with proceeded time, they became two distinct specialties with not adequate scientific collaboration [51]. Epilepsy is one of the most common neurological disorders with a high prevalence of multiple psychiatric comorbidities (up to 50%) suggesting the presence of a shared common pathophysiology [54]. These comorbidities are extremely important especially for patients with drug-resistant epilepsy and epilepsy surgery candidates [55]. Psychiatric comorbidities are accepted to be an independent predictor of seizure outcome in surgery candidates, with considerably decreased seizure-freedom rates after surgery, in the presence of psychiatric disorders [56]. Therefore, epilepsy-specific psychological interventions should be a part of routine evaluation and a multidisciplinary team work should be implanted to the treatment to increase quality of life in these patient groups [57]. Physicians always should be aware of the additional burden of psychic disorders in drug-resistant epilepsy patients.

For all disciplines to work in harmony for the diagnosis, differential diagnosis, treatment strategies, and follow-up of patients with epileptic disorders, further research for guidelines-based care and improved opportunities for PCPs and psychiatrists and psychologists to consult with neurologists is warranted [58].

#### 5.2.4. Agreement

A.Ç.A.’s interpretation aligns with established medical consensus on accurate seizure diagnosis. The consideration of psychogenic episodes resonates with the broader medical framework, while the subsequent differentiation based on clinical indicators echoes established practice.

#### 5.2.5. Disagreement

While A.Ç.A. proposed invasive methods for identifying the seizure onset, practical challenges, patient reluctance, and ethical considerations may limit the feasibility of such approaches.

#### 5.2.6. Reflection

The presented case underscores the intricate process of diagnosing refractory seizures. Clinical indicators, invasive procedures, and multimodal assessments interplay to achieve a holistic diagnosis.

#### 5.2.7. Education (Key Messages)

- Multidisciplinary approach is mandatory when evaluating a patient with seizures;
- Primary care physicians are the initial referments for the patients with new onset seizures;
- Psychiatric evaluation is very important in the care of patients with epilepsy especially in terms of treatment selections for these patients;
- Further research for guidelines-based care and improved opportunities for PCPs and psychiatrists/psychologists to consult with neurologists is warranted.

### 5.3. Psychiatric Point of View by F.İ.

#### 5.3.1. Comprehension

In this context, we delved into a case study that highlighted the intricate relationship between epilepsy and psychiatric symptoms. My appraisal offers insights into the challenges of distinguishing epilepsy-related symptoms from psychiatric manifestations.

#### 5.3.2. Appraisal

Epilepsy symptoms can overlap with certain psychiatric symptoms, and individuals with epilepsy can also experience psychiatric comorbidities. This overlap sometimes makes it challenging to clinically distinguish between epilepsy and psychiatric symptoms in these patients. The case we are discussing involves early onset epilepsy and autism-like findings. The onset of chronic diseases like epilepsy in childhood often leads to lengthy hospital stays and treatment searches, significantly impacting a child’s life. Stigmatization of epilepsy patients among their peers during childhood can persist into adulthood, resulting in a high level of perceived stigma among children and adolescents with epilepsy [59]. This stigma has severe debilitating effects, affecting school attendance, quality of life, self-esteem, social interaction, and performance. Consequently, it can influence an individual’s development and personality traits during adulthood. In our case, the presence of autism-like symptoms, dependency on the family, and functional impairments made us consider the psychiatric dimensions of the disease. Moreover, our case had epilepsy seizures that were resistant to treatment, and the accompanying autism spectrum features may increase the likelihood of additional psychiatric diseases. Additionally, epilepsy seizures are often mistaken for conversion seizures, but it is important to note that conversion seizures can also occur in individuals with epilepsy [59]. The best clinical distinction from a psychiatric perspective can be achieved through a detailed psychiatric examination, assessment of disease history, and regular clinical follow-ups. For patients in this group, a comprehensive neurological and psychiatric evaluation is necessary.

#### 5.3.3. Research

Epilepsy is not solely characterized by seizures but also represents a brain disorder that can result in neurological, cognitive, and psychosocial consequences. Psychiatric comorbidities frequently accompany epilepsy [60]. These psychiatric conditions can exist prior to the onset of epilepsy, act as the cause or consequence of epilepsy, and occur at any time during the disorder [61]. Common psychiatric disorders in epilepsy include depression, anxiety disorders, psychosis, personality changes, cognitive abnormalities, and attention deficit. Unfortunately, psychiatric disorders often go unnoticed in epilepsy patients as seizure control becomes the primary focus of management [62]. Clinicians may not be aware that psychiatric disorders can occur in individuals with epilepsy. However, psychiatric comorbidities can lead to poor treatment response, impact the patient’s quality of life, and increase the risk of early death due to suicide or accidents [63]. Therefore, it is crucial to identify and consider psychiatric diseases that accompany epilepsy in treatment planning [64]. These patients should be evaluated by a multidisciplinary team for accurate diagnosis and comprehensive treatment.

#### 5.3.4. Agreement

My observations resonate with the broader medical understanding of epilepsy’s multifaceted impact. The recognition of psychiatric dimensions in epilepsy underscores the need for holistic evaluations that encompass both neurological and psychiatric perspectives.

#### 5.3.5. Disagreement

While my advocated for comprehensive psychiatric assessments, practical challenges, limited resources, and the availability of specialized professionals may hinder the widespread implementation of such evaluations.

#### 5.3.6. Education (Key Messages)

- Epilepsy and psychiatric comorbidity are frequently observed together;
- Psychiatric diseases accompanying epilepsy can significantly affect the prognosis and functional outcomes of the disease;
- The treatment and follow-up of epilepsy require the involvement of a multidisciplinary team;
- It is important to recognize and address psychiatric comorbidities in individuals with epilepsy to improve their quality of life and treatment outcomes;
- Comprehensive evaluation and treatment of epilepsy should involve collaboration among neurologists, psychiatrists, and other healthcare professionals.

### 5.4. Family Physician Point of View by N.T.

#### 5.4.1. Comprehension

In this exploration, we examined the critical role of family physicians in the management of epilepsy. My appraisal emphasized the need for a biopsychosocial approach, collaborative care, and combating stigma associated with epilepsy.

#### 5.4.2. Appraisal

Primary care is the first point of contact for individuals experiencing epileptic seizures, particularly in developed countries with established referral chains [65]. The biopsychosocial approach is a fundamental principle in family medicine, although its progress has been slow due to the prevailing biomedical perspective. This model, initially developed by George Engel, aids family physicians in understanding the interplay between biological and psychosocial factors in illness and facilitates the development of multidisciplinary approaches to patient care. It has been suggested that the biopsychosocial model has the potential to improve clinical outcomes for chronic diseases and functional disorders encountered in primary care, including epilepsy [66].

There have been reports of an adolescent with epilepsy facing stigmatization at school, leading to an increase in seizure frequency after being excluded from certain activities. Epilepsy is often perceived by society as dangerous and frightening, and these misconceptions persist even when seizures are effectively controlled through medication [67]. Family physicians play a crucial role in combating such stigma.

#### 5.4.3. Research

Several reports indicated that only 17% of patients with new-onset epileptic seizures in the United States receive neurological evaluations. While family physicians are important in the care of individuals with seizures, epilepsy is a complex condition that requires specialized knowledge for accurate diagnosis, classification, and treatment. Consequently, family physicians may not always be aware of the latest diagnostic and therapeutic approaches [68]. There is a need to enhance collaboration between family physicians and related specialists, particularly neurologists, for differential diagnosis and ongoing management. Treatment decisions, including the choice of antiepileptic drugs, depend on factors such as seizure type, side effect profiles, and comorbidities. Guidelines defining seizure characteristics and criteria for referral should be disseminated to ensure optimal care [69]. Despite the availability of medications, long-term adherence to treatment among individuals with epilepsy remains a challenge. Some suggest that home care services may improve treatment adherence, seizure control, and quality of life for these individuals. This highlights the importance of comprehensive follow-up beyond medication management [70]. To support the proactive role of family physicians in epilepsy care, further research is needed. Establishing interdisciplinary collaborative relationships and recognizing the expertise and shared goals of relevant fields will benefit patients. This approach can contribute to providing the best possible medical care for individuals with epilepsy in the present era [68]. Family physicians have a significant responsibility in combating epilepsy-related stigma.

#### 5.4.4. Agreement

Our perspective aligns with the broader healthcare ethos of promoting collaboration among specialties for comprehensive patient care. Recognizing the limitations of family physicians in managing complex conditions like epilepsy, the call for enhanced collaboration with neurologists is essential.

#### 5.4.5. Disagreement

While I have emphasized the need for family physicians to stay updated with the latest diagnostic and therapeutic approaches, practical constraints such as time limitations and access to specialized training may hinder their ability to fully engage with complex conditions like epilepsy.

#### 5.4.6. Reflection

This discussion compels us to reflect on the profound impact of epilepsy-related stigma on patients’ lives. It reinforces the importance of family physicians not only as medical caregivers but also as advocates who can actively combat stigma through patient education and support.

#### 5.4.7. Education (Key Messages)

- Epilepsy is a growing global public health concern, requiring family physicians to take a more proactive role;
- Family physicians are vital in addressing epilepsy-related healthcare needs;
- Guidelines should be available to family physicians, providing criteria for seizure characteristics and appropriate referral [69];
- Training family physicians to advocate for patients and prevent epilepsy-related stigma is essential;
- Comprehensive follow-up and collaboration among healthcare disciplines contribute to optimal epilepsy management in the modern healthcare landscape.

## 6. Case 4: Bipolar Affective Disorder with Normal Pressure Hydrocephalus

### 6.1. Summary of Cases by S.Y.Ç.

A 66-year-old married woman presented with a range of complaints over the past month, including decreased sleep, decreased appetite, increased speed and quantity of speech, irritability, forgetfulness, and inappropriate urination. Within the last week, she developed additional symptoms, such as a belief that she would be poisoned by meals prepared at home, a sense of grandiosity, and aggression, which prompted her family to bring her to our emergency department. During the psychiatric examination, the patient exhibited irritability, increased speech rate, and paranoid and persecutory delusions. Neurological examination did not reveal any abnormal findings except for magnetic gait.

In her medical history, it was noted that the patient had been diagnosed with bipolar affective disorder 30 years ago and had been in remission with medication compliance until 12 years ago. At that time, she was diagnosed with normal pressure hydrocephalus (NPH) following her referral to neurology due to gait disturbance and urinary incontinence. Subsequently, she underwent ventriculoperitoneal shunt surgery. The patient’s most recent psychiatric hospitalization occurred three years ago, and her medication compliance has been poor for the past year.

Upon admission, the patient’s Young Mania Rating Scale (YMRS) score was evaluated as 39. To address her agitation and acute psychotic symptoms, she was started on Haloperidol 10 mg/day and biperiden 2.5 mg/day intramuscularly. Once her agitation subsided, a Mini-Mental State Examination was performed, revealing a score of 14/30. Considering her medical history, an MRI was conducted, and consultations were sought from the neurology and neurosurgery departments. The MRI images indicated an Evans index of 0.41 and a Callosal angle of 107 degrees. It was determined that there was no dysfunction in the shunt, and based on the patient’s current symptoms, a follow-up with the neurology department was recommended.

Given the persistence of the patient’s affective symptoms, a treatment plan was established, which included Valproic acid 500 mg/day and Olanzapine 5 mg/day. In the second week, the dosage of Valproic acid was increased to 1000 mg/day, and Olanzapine was raised to 20 mg/day. Additionally, Solifenacin succinate 5 mg/day and Piracetam 2400 mg/day were added to address urinary incontinence, and monitoring for dementia was advised. During the 6th and 7th-week follow-ups, the patient’s Young Mania Rating Scale (YMRS) score improved to 18, and there were no changes in the Mini-Mental State Examination. Consequently, the patient was discharged.

### 6.2. Neurological Point of View by A.Ç.A.

#### 6.2.1. Comprehension

This analysis delved into the intricate connection between bipolar affective disorder and NPH S.Y.Ç. provided a comprehensive overview, highlighting the relevance of organic etiologies in psychiatric presentations.

#### 6.2.2. Appraisal

This case report emphasizes the importance of considering an organic etiology in patients presenting with psychiatric symptoms, such as bipolar disorder in this case. The patient had been diagnosed with bipolar disorder 30 years ago and had been in remission for the past 12 years. It remains unclear whether the coexistence of normal pressure hydrocephalus (NPH) and bipolar disorder in this patient is due to a shared underlying pathophysiology or simply coincidental. Notably, the patient’s psychiatric symptoms showed resistance but responded to high doses of antipsychotic and mood stabilizing agents. The persistence of these symptoms raised concerns regarding a possible dysfunction of the patient’s ventriculoperitoneal shunt, but the shunt was found to be functioning properly. The patient’s low MMSE score alerted the clinicians, leading to monitoring by both the neurology and psychiatry departments to assess the possibility of a new onset degenerative dementia, such as Alzheimer’s disease. It is important to differentiate symptoms of dementia in NPH from other types of dementia, as the former can be reversible with early treatment, whereas symptoms of neurodegenerative dementia are irreversible [71]. Therefore, repeated neuropsychological tests during follow-up are crucial for detecting the potential development of neurodegenerative dementia, such as Alzheimer’s disease, in this patient. The early diagnosis and treatment of NPH require the collaboration of multiple disciplines, including neurosurgeons, neurologists, psychiatrists, and urologists [72].

#### 6.2.3. Research

Idiopathic NPH (INPH) is characterized by a triad of gait disturbance, dementia, and urinary incontinence, accompanied by dilation of the ventricular system with normal opening cerebrospinal fluid pressure [73]. The prevalence of INPH among patients older than 65 years is estimated to be between 0.3% and 3% and increases with age [74]. It is a progressive but potentially treatable disease, and the only effective treatment is CSF shunting [75]. Diagnosis can be challenging as all three elements of the diagnostic triad may not be present simultaneously, and neurological examination may show subtle clues. Therefore, the diagnosis should be supported by the presence of ventriculomegaly (Evans index ≥ 0.3 in magnetic resonance imaging) and the absence of obstruction to CSF flow on neuroimaging [76].

Normal pressure hydrocephalus has been associated with various psychiatric manifestations, such as schizophrenia, apathy, anxiety, depression, etc., although most of the data come from case reports and the prevalence is estimated to be low [75]. The underlying pathophysiology of cognitive and psychiatric disruption in NPH is still not fully understood, but dysfunction of the frontal lobe, along with reduced volumes of subcortical deep gray matter nuclei, is believed to play a role [77]. Additionally, changes in intracerebral pressure may contribute to neuronal damage and affective symptoms [78].

Due to the similarity of clinical symptoms, NPH is often misdiagnosed as Alzheimer’s disease. A comprehensive diagnostic workup, including detailed history taking, neuropsychological examinations, MRI, and other diagnostic interventions, should be performed to differentiate between these conditions [71]. Given the wide range of clinical manifestations of NPH, which can mimic other neurodegenerative and psychiatric disorders, adherence to structured guidelines is essential for achieving a definitive diagnosis [79].

#### 6.2.4. Agreement

S.Y.Ç.’s perspective aligns with a broader trend of understanding the complex interplay between mental health and organic conditions. The case encourages clinicians to consider organic etiologies in patients presenting with psychiatric symptoms.

#### 6.2.5. Disagreement

While S.Y.Ç. provided a comprehensive assessment of the patient’s history, symptoms, and treatment, it is important to acknowledge that the specific relationship between NPH and bipolar disorder remains a topic of ongoing research, with potential factors that could contribute to their co-occurrence yet to be fully elucidated.

#### 6.2.6. Reflection

This examination prompted us to reflect on the critical importance of thorough diagnostic assessments. The case study reminded us that the overlapping symptomatology of different conditions can complicate accurate diagnosis, necessitating a multidisciplinary approach and adherence to structured guidelines.

#### 6.2.7. Education (Key Messages)

- NPH is a progressive but potentially treatable disease, and the only effective treatment is CSF shunting;
- The wide range of clinical manifestations of NPH can mimic other neurodegenerative and psychiatric disorders, highlighting the importance of following structured guidelines;
- Early diagnosis and treatment of NPH require the collaboration of multiple disciplines, including neurosurgeons, neurologists, psychiatrists, and urologists.

### 6.3. Psychiatric Point of View by E.E.B. and S.Y.Ç.

#### 6.3.1. Comprehension

This analysis dived into the intricate interplay between bipolar disorder and various neurological conditions, offering insights into their mutual impacts and shared complexities.

#### 6.3.2. Appraisal

Bipolar disorder is one of the common psychiatric disorders that affect the entire population. When left untreated, it can lead to negative outcomes such as early death and deterioration of overall health. However, when correctly diagnosed and appropriately treated, it is possible to improve the patient’s quality of life [80]. As individuals with bipolar disorder (BD) age, it is observed that the comorbidity with other psychiatric disorders decreases. However, there is some evidence indicating that neurological and other somatic disorders are more prevalent in elderly bipolar patients compared to the general elderly population [81]. When comparing elderly patients with BD to elderly patients with unipolar depression, Shulman and colleagues found that 36% of bipolar patients had a neurological disorder in old age, whereas only 8% of patients with recurrent depressive disorder had one [82]. This may be attributed to different etiologies such as vascular, traumatic, or degenerative causes. In general, the presence of manic symptoms in elderly individuals predicts a worse course compared to depressive symptoms alone [83]. A better understanding of late-life bipolar disorder can lead to more specific recommendations for tailored treatment based on the specific features and needs resulting from age-related somatic and cognitive changes [84]. In certain cases, secondary mania can occur. Cerebrovascular disease, dementia, epilepsy, brain tumors, and encephalitis are among the neurological causes that can lead to mania. Approximately 10% of patients who have experienced closed head trauma exhibit manic symptoms within the first year following the trauma [85]. Vascular dementia, Huntington’s disease, normal-pressure hydrocephalus, and prion diseases have also been associated with mania [7]. Systemic causes that can contribute to mania include Cushing’s syndrome, hyperthyroidism, and vitamin B12 deficiency [86].

#### 6.3.3. Research

Normal pressure hydrocephalus is a syndrome characterized by gait disturbance, dementia, urinary incontinence, and ventricular system dilation due to impaired CSF circulation with normal CSF pressure [86]. It is noted that NPH patients may exhibit specific neuropsychiatric symptoms that could be associated with alterations in central neurotransmitter activity [87].

NPH patients can develop symptoms with frontal dominance, such as personality changes, anxiety, depression, delusional states, hallucinations, apathy, and aggressive behavior. Rare cases of obsessive–compulsive disorder, Othello syndrome, and mania have been reported in the literature [87,88]. Cognitive impairment in NPH can manifest as mild to moderate dementia [89]. Impaired attention, executive functions, and psychomotor slowing have been described as the earliest cognitive deficits in NPH [90].

Among the most observed neuropsychiatric symptoms in NPH, apathy is known to be the most prevalent, followed by anxiety and aggression. Anxiety, agitation, and stereotypical behavior have been reported in 25%, 17%, and 14% of NPH patients, respectively [91]. Comorbid conditions in bipolar patients may play a role in the resistance of episodes. The emergence of serious side effects with low-dose antipsychotics should prompt clinicians to consider the possibility of underlying organic conditions.

#### 6.3.4. Agreement

The perspective aligns with the overarching trend towards comprehensive care, acknowledging the multifaceted nature of psychiatric and neurological interactions. The analysis emphasized that a nuanced understanding is vital for tailored treatment.

#### 6.3.5. Disagreement

While the appraisal discussed various potential causes of neurological symptoms in bipolar patients, it is important to recognize that the etiological relationship between bipolar disorder and these conditions is complex and often involves a bidirectional influence.

#### 6.3.6. Reflection

This examination encouraged us to reflect on the intricate relationships between psychiatric and neurological disorders. The insights presented prompt us to approach diagnosis and treatment with heightened awareness of the potential interplay.

#### 6.3.7. Education (Key Messages)

- In elderly individuals with bipolar disorder, it is crucial to assess and monitor for the presence of comorbidities such as cardiovascular diseases, diabetes, metabolic syndrome, thyroid disorders, substance-use disorders, anxiety disorders, and neurocognitive disorders (e.g., dementia). This highlights the need for comprehensive evaluation and tailored treatment approaches considering age-related changes;
- Comorbidity can indeed affect the treatment response of bipolar disorder, increase the severity of symptoms, and complicate the treatment process;
- Bipolar disorder is a prevalent psychiatric disorder that can significantly impact a person’s overall health and quality of life if left untreated. Proper diagnosis and appropriate treatment are essential for improving outcomes and managing the condition effectively;
- Secondary mania can occur due to various neurological causes such as cerebrovascular disease, dementia, epilepsy, brain tumors, closed head trauma, and certain systemic conditions. Identifying and addressing these underlying organic conditions is crucial for managing manic symptoms in bipolar patients and optimizing treatment outcomes’
- Apathy is the most prevalent neuropsychiatric symptom in NPH, followed by anxiety and aggression. Understanding and addressing these symptoms in NPH patients can contribute to their overall management and treatment plan.

### 6.4. Family Physician Point of View by N.T.

#### 6.4.1. Comprehension

This perspective delves into the escalating prevalence of psychiatric issues, with a spotlight on bipolar disorder, highlighting the role of family physicians in early recognition, treatment, and follow-up. It underscores the importance of a holistic approach, integrated care, and proactive management of comorbidities.

#### 6.4.2. Appraisal

Currently, the prevalence of psychiatric problems is rapidly increasing, highlighting the significance of this issue as a public health concern. Bipolar disorder, one of the most prevalent psychiatric disorders, poses substantial risks of disability when left untreated. Therefore, it is crucial for all healthcare professionals to be attentive to bipolar disorder in terms of early recognition, acute phase management, follow-up, and treatment. By emphasizing treatment compliance and improving the quality of life and social functioning of patients, healthcare providers can enhance patient outcomes [92].

In family health centers, which serve as the primary point of contact for individuals seeking healthcare, it is essential to carefully evaluate recurrent psychiatric symptoms and referral criteria. In accordance with the WONCA European definition, a “holistic approach” should be adopted, encompassing the utilization of a biopsychosocial model that considers multiple dimensions [93].

Primary care settings, due to the nature of the services provided, offer a more favorable environment for delivering holistic care compared to institutional settings. Family physicians, as part of their responsibilities, also offer mental health care, thus providing integrated and comprehensive services to patients [94]. The long-term follow-up of patients with bipolar disorder is typically conducted in family medicine. Since most patients experience periodic relapses, it is especially crucial to facilitate their transition back to the home environment after psychiatric hospitalization or for other reasons. This process carries inherent risks and necessitates close attention and support, with primary healthcare services playing a vital coordinating role. In addition to social support, treatment regimens should be balanced to ensure successful reintegration into the workforce [95].

#### 6.4.3. Research

Research has shown that individuals with bipolar disorder often experience comorbidities, particularly metabolic and marital disorders. It is crucial to regularly screen for and closely manage such conditions to prevent their associated high morbidity and mortality rates [96]. Primary care physicians can offer comprehensive medical care by educating and supporting patients and their families, as well as diagnosing and treating bipolar disorder and comorbid conditions. Determining the role of family physicians in managing patients with bipolar disorder and providing appropriate counseling, referral, and support services when needed is of utmost importance. Further studies are needed to explore potential approaches in this direction within the existing literature [95].

#### 6.4.4. Agreement

The perspective aligns with the ongoing shift toward integrating mental health care within primary care services. It supports equipping family physicians with the skills and awareness to effectively address bipolar disorder, comorbidities, and the various dimensions of patient care.

#### 6.4.5. Disagreement

While the appraisal highlighted the suitability of primary care for delivering holistic care, potential challenges in resource availability, time constraints, and specialized expertise might limit the extent to which family physicians can address all facets of bipolar disorder management.

#### 6.4.6. Reflection

This analysis prompted reflection on the multifaceted responsibilities of family physicians in managing bipolar disorder. It encouraged us to consider how these practitioners can effectively balance their roles in diagnosis, treatment, referral, and coordinating patient car

#### 6.4.7. Education (Key Messages)

Enhance family physicians’ diagnostic skills and management of bipolar disorder through sensitizing training.

- Promote patient and family education and support programs to foster better understanding and management of bipolar disorder;
- Establish cooperative initiatives to facilitate post-hospital follow-up in primary care settings;
- Highlight the pivotal role of family physicians in managing comorbid conditions through meticulous patient records and long-term follow-up.

## 7. Case 5: Postherpetic Neuralgia (PHN) in a Case with Bipolar Affective Disorder

### 7.1. Summary of Cases by N.Ö.

A 66-year-old female patient presented at our clinic with a complaint of neuropathic pain in the right breast persisting for 3 months. The pain was described as unbearable, characterized by stinging and burning sensations that began concurrently with the development of a vesicular rash. The patient had previously been diagnosed with shingles, and treatment with oral acyclovir and cream had been initiated, but there was no improvement in the pain. She experienced severe pain radiating from the breast to the armpit and back, including the right nipple. The pain worsened with arm movement and was accompanied by allodynia and hyperalgesia. The patient rated her pain on the Visual Analog Scale (VAS) as 10. During the examination, the patient exhibited moderate hypomimia, bilateral symmetrical bradykinesia, and rigidity in all extremities. She had a slight stooped posture but did not show any loss of postural reflexes. There were no pyramidal signs or associated findings such as restricted gaze or frequent falls. In terms of her medical history, she had a history of Lithium use for bipolar disorder, as well as diabetes mellitus, hypertension, and drug-induced parkinsonism. There were no significant features in her family history. She was currently taking Levodopa-Benserazide and Lithium medications, with normal lithium levels (1.1 meq/lt). The patient was diagnosed with post-herpetic neuralgia, and treatment with Pregabalin was initiated. The dose of pregabalin was gradually increased to the maximum recommended dosage. The patient also underwent dorsal root ganglion radiofrequency treatment, which was assessed by a simultaneous algology evaluation. In addition, opioids and Duloxetine were introduced; however, despite these treatments, only partial improvement in the patient’s pain was observed. Subsequently, the patient received 100 units of Onabotulinum toxin A (BoNT A), and after the initial application, her pain was reduced by half. Following the administration of the second dose 15 days later, the patient experienced an approximately 80% improvement in her condition.

### 7.2. Neurological Point of View by E.A.D.

#### 7.2.1. Comprehension

This analysis offered a comprehensive understanding of a complex case involving PHN in a patient with bipolar disorder. It highlighted the challenges of managing refractory pain, explored various treatment modalities, and emphasized the importance of multidisciplinary collaboration.

#### 7.2.2. Appraisal

The patient has refractory PHN, which refers to pain persisting after an acute episode of herpes zoster beyond the healing of the rash [97]. This pain is typically localized and one-sided, presenting with itching, burning, sharp, stabbing, or throbbing sensations [98]. Some patients may also experience allodynia, hyperalgesia, and deficits in thermal, tactile, pinprick, or vibration sensations within or beyond the affected areas of the skin, as observed in this patient [99]. These symptoms can last for months or even years, significantly impacting a patient’s quality of life.

In the case of this patient, the pain has persisted for three months. Initially, PHN is typically treated with medication, including first- and second-line therapies such as tricyclic antidepressants, serotonin-norepinephrine reuptake inhibitors, pregabalin, gabapentin, tramadol, capsaicin (8%) patches, and lidocaine patches [100]. The patient received antiviral medication, which resolved the rash but did not alleviate the pain. Despite maximum doses of pregabalin, duloxetine, opioids, and compounded lidocaine cream, the patient did not experience any improvement in their pain.

Dorsal root ganglion (DRG) radiofrequency was then applied to the patient after evaluation by an algologist. Pulsed radiofrequency application to the DRG has shown effectiveness in treating treatment-resistant cases of herpes zoster and PHN [101]. However, the patient did not respond to this treatment either. Due to the lack of response to previous treatments, Ona botulinum toxinA therapy was administered to the patient. Following this treatment, the patient experienced an approximately 80% improvement in their condition. Limited reports exist on the long-term safety and efficacy of BoNT A therapy for severe chronic pain in PHN cases [102].

Additionally, the patient was using lithium for bipolar disorder and experienced drug-induced parkinsonism. Although lithium is a mood stabilizer, it is rarely associated with drug-induced parkinsonism, even at therapeutic serum levels [103]. Close monitoring of lithium levels in geriatric populations is important, and when parkinsonism symptoms appear in chronic lithium users, the possibility of lithium-induced parkinsonism should be considered [103].

#### 7.2.3. Research

PHN is a common cause of neuropathic pain resulting from infection by the herpes zoster virus. Approximately 12.5% of individuals with zoster over the age of 50 develop PHN. The incidence of PHN increases with age and is more prevalent in individuals with diabetes mellitus. PHN is characterized by severe neuropathic pain that occurs following nerve injury. The nerve injury involves processes such as demyelination, axonal loss, degeneration of small nerve fibers, reorganization in the spinal cord’s dorsal horn, and neuroplastic changes [102].

Despite various treatment approaches, PHN often proves to be refractory. The efficacy of administering antiviral medication at the onset of the rash to reduce the incidence of PHN remains inconclusive. Medication is typically the initial treatment for PHN. The European Federation of Neurological Societies provides Level A evidence supporting the use of both first- and second-line medications, including tricyclic antidepressants, serotonin-norepinephrine reuptake inhibitors, pregabalin, gabapentin, tramadol, capsaicin (8%) patches, and lidocaine patches [103].

Pulsed radiofrequency (PRF) application to the dorsal root ganglion (DRG) has proven to be a beneficial treatment for cases of herpes zoster and PHN that do not respond to other interventions. The DRG contains numerous receptor channels and plays a crucial role in nociceptive signaling. Targeting the DRG is thus a priority for managing zoster-related pain. Studies have reported the effects of PRF on the DRG in patients with PHN [101,104].

Limited reports exist on the safety and effectiveness of long-term BoNT A therapy for severe chronic pain in PHN, particularly in cases where oral medication is ineffective or not tolerated. BoNT A appears to be a promising option for long-term management of severe PHN, offering significant pain reduction for up to 3–4 months per injection cycle. Combining BoNT A injections with standard oral medication yields greater pain reduction in PHN. Recommended doses range from 5 to 10 IU per injection point, and a higher number of injection points per session may result in longer-lasting pain reduction [102].

#### 7.2.4. Agreement

The perspective resonates with the necessity of a multidisciplinary approach in addressing complex cases involving comorbidities such as bipolar disorder and refractory PHN. It emphasizes that coordinating efforts from various healthcare professionals is crucial for comprehensive patient care.

#### 7.2.5. Disagreement

While the analysis highlighted the association between lithium use and drug-induced parkinsonism, it is important to acknowledge that the link is rare and typically observed at therapeutic serum levels. Caution should be exercised when generalizing this association to all cases of lithium use.

#### 7.2.6. Reflection

This examination prompted reflection on the intricacies of managing refractory pain while considering comorbid conditions. It encouraged us to explore the delicate balance between pain relief strategies and the impact of these treatments on other existing medical conditions.

#### 7.2.7. Education (Key Messages)

- PHN is a condition that leads to severe pain and disability;
- It is important to be cautious and monitor for manic symptoms when initiating treatment with SNRIs in patients with bipolar disorder;
- A multidisciplinary approach is essential when assessing patients with psychiatric comorbidities, as it requires input from various healthcare professionals;
- Primary care physicians should exercise caution and pay attention to potential drug interactions in patients who are taking multiple medications.

### 7.3. Psychiatric Point of View by E.E.B.

#### 7.3.1. Comprehension

This evaluation provided insight into the complexities of treating bipolar disorder, focusing on the importance of continuity of care and long-term follow-up. It underscores the role of lithium in treatment, its potential complications, and the consideration of antipsychotic combination therapy.

#### 7.3.2. Appraisal

Continuity of care in follow-up visits within primary healthcare services is crucial for patients with bipolar disorder, especially considering the long-term nature of their maintenance drug therapy. These treatments can persist for years or even a lifetime. It is estimated that approximately half of the patients who recover from a mood episode will experience a recurrent episode within two years. Exacerbations in bipolar patients can lead to higher rates of suicide attempts, neurodegeneration, and increased social and occupational problems. Therefore, ensuring effective maintenance treatment through long-term follow-up is vital. Effective maintenance therapy can help prevent or delay further exacerbations, ultimately minimizing disease progression and improving cognitive impairment. Lithium has been a significant pharmacological treatment for recurrent bipolar disorder for many years, demonstrating high efficacy. However, it also carries the risk of multisystemic adverse effects and toxicities, which can potentially lead to serious and life-threatening complications [105]. To enhance the speed of therapy and reduce neuronal burden, lithium treatment is often combined with at least one antipsychotic drug in more than 50% of patients. This combination can induce extrapyramidal symptoms, such as imbalances between the dopaminergic and cholinergic systems. Lithium can override dopamine in the rat striatum and limbic forebrain. Extrapyramidal symptoms, such as stretching, orofacial or whole-body movements, and cogwheel rigidity, can occur with lithium alone or after long-term exposure to antipsychotics. Close and regular clinical observation, along with monitoring lithium serum levels, is necessary to minimize potential complications. However, it is important to note that serum levels may not always accurately reflect intracellular concentrations and that adverse effects may persist even within the normal range. Considering the potential risk of drug interactions and cumulative effects when combining lithium with antipsychotics, it is essential to be mindful of these factors during treatment [106].

#### 7.3.3. Research

PHN is a significant complication of Herpes Zoster (HZ), particularly affecting older individuals and those with weakened immune systems. Despite advancements in PHN treatments, it continues to have a negative impact on the quality of life for many people. Notably, the Zoster vaccine has been shown to significantly reduce the incidence of both HZ and PHN. Early diagnosis and prompt treatment of HZ can help alleviate the burden of PHN [107]. It is acknowledged that the management of neuropathic pain associated with PHN often falls short with current treatment approaches. The challenges include complex and cumbersome treatment regimens, potential side effects of medications, the presence of comorbid conditions, and the use of multiple medications. There is a need for comprehensive evaluation and dissemination of information on the available pharmacological treatment options to ensure effective pain control, especially in primary care settings [108].

#### 7.3.4. Agreement

The perspective aligns with the need for careful management of bipolar disorder, especially when using lithium. It underscores the efficacy of lithium while acknowledging its potential for multisystemic adverse effects and the importance of vigilant monitoring.

#### 7.3.5. Disagreement

While the analysis discussed the occurrence of extrapyramidal symptoms in lithium-antipsychotic combination therapy, it is important to acknowledge that these symptoms primarily arise due to antipsychotic medications’ interaction with the dopaminergic system.

#### 7.3.6. Reflection

This examination prompted reflection on the balance between expedited therapy and potential side effects. It encourages healthcare providers to approach treatment with careful consideration of medication interactions and adverse effects.

#### 7.3.7. Education (Key Messages)

- Continuity of care and long-term follow-up in primary healthcare settings are crucial for patients with bipolar disorder to ensure effective maintenance treatment and prevent recurrent mood episodes;
- Lithium is an effective pharmacological treatment for recurrent bipolar disorder, but careful monitoring is necessary due to the potential for multisystemic adverse effects and toxicities;
- Combining lithium with antipsychotic drugs can enhance the speed of therapy, but it may increase the risk of extrapyramidal symptoms. Regular clinical observation and monitoring of lithium serum levels are essential;
- The Zoster vaccine is recommended for the prevention of PHN, particularly in the elderly population;
- Early diagnosis and prompt treatment of Herpes Zoster can help alleviate the burden of PHN, emphasizing the importance of effective pain control for PHN through comprehensive evaluation and dissemination of pharmacological treatment options in primary care settings.

## 8. Case 6: Neurosyphilis Presenting with Psychotic Symptoms

### 8.1. Summary of Cases by Y.S.F.

A 23-year-old unmarried female patient, who is a first-year university student, presented with complaints that have been ongoing for approximately 1 year. During this time, the patient’s family reported that she has become withdrawn and unable to attend school. Over the past two months, additional symptoms have emerged, including talking to herself, feeling paranoid and believing she is being watched, and experiencing irritability. Following a suicide attempt by jumping from a height, her family brought her to the emergency department of our hospital.

During the mental state examination, the patient exhibited positive findings, including auditory hallucinations, paranoid delusions, and a sense of detachment from reality (derealization). The physical examination did not reveal any notable abnormalities. The patient’s past medical history revealed that she had experienced anxiety symptoms three years ago after breaking up with her boyfriend. This was followed by a period of benzodiazepine abuse and subsequent use of multiple psychoactive substances. Her most recent substance use occurred eight months ago with methamphetamine, and she has not used any psychoactive substances since then. The patient’s family also mentioned that they had not heard from her for a few days two years ago.

After being admitted to our inpatient clinic, laboratory tests were conducted. Serologic investigations of her serum showed positive results for both the venereal disease research laboratory rapid plasma reagin test (VDRL-RPR) and the treponema pallidum hemagglutination assay (TPHA), with serum titers of 1/32 and 1/1280, respectively. A lumbar puncture (LP) was planned for further evaluation. In the meantime, based on the recommendation of the infectious diseases department, the patient was initiated on a weekly dose of 2.4 million units of penicillin G benzathine as treatment until the LP results were obtained. Risperidone was also started to address the patient’s positive psychotic symptoms and gradually increased to a dosage of 4 mg per day.

After the LP, CSF analysis revealed a white blood cell count of 20/mm^3^, glucose level of 58 mg/dL, protein level of 0.55 g/L, and a positive TPHA test with a titer of 1/16. Both the CSF and serum assessments did not indicate any signs of specific infections or viral diseases, including HIV. Following consultation with the infectious diseases department regarding the LP results, it was determined that the patient requires close monitoring and should be transferred to the infectious diseases service for further management and administration of procaine penicillin treatment.

### 8.2. Neurological Point of View by A.Ç.A.

#### 8.2.1. Comprehension

This analysis delved into a case involving a young woman with emergent psychotic symptoms and a history of substance abuse. It emphasized the importance of considering organic causes and conducting a comprehensive diagnostic work-up.

#### 8.2.2. Appraisal

This is a case of a young, sexually active adult who had not been diagnosed with any psychosis before but has gradually developed psychotic symptoms over time. It is important for the clinician to be aware of the patient’s history of benzodiazepine and multiple psychoactive substance abuse, as this suggests the possibility of an organic cause for her symptoms. In a patient experiencing their first psychotic episode without any previous psychiatric illness, it is crucial to conduct a comprehensive differential diagnosis to rule out any organic causes. This includes considering inherited disorders such as Huntington’s disease, Wilson’s disease, acute intermittent porphyria, neuroacanthocytosis, etc. Additionally, neurodegenerative disorders in the elderly population, such as frontotemporal dementia, REM sleep behavior disorder preceding the motor onset of Parkinson’s disease, Lewy body disease, and Alzheimer’s disease, should be considered. Vascular diseases like acute stroke, certain forms of epilepsy (particularly temporal lobe or frontal lobe epilepsies), infectious and inflammatory diseases (such as syphilis, Creutzfeldt-Jakob disease, and Systemic Lupus Erythematosus), deficiency disorders (like Wernicke’s encephalopathy and vitamin B12 deficiency), malignancies such as frontal lobe tumors, head trauma, and thyroid function disorders should also be included in the differential diagnosis.

In addition to obtaining a detailed patient history (including sexual activity, drug abuse, travel history, etc.), a comprehensive diagnostic work-up should be performed. This may include blood analysis (complete blood count, erythrocyte sedimentation rate, comprehensive metabolic panel, thyroid function tests, specific tests for suspected infectious agents), a thorough physical examination, detailed neurological examination, cognitive assessment, neuroimaging (such as cranial magnetic resonance imaging), and, if necessary, lumbar puncture and electroencephalography. Given that many central nervous system inflammatory and infectious diseases can present with neuropsychiatric symptoms, it is crucial to consider the possibility of an underlying organic cause in order to avoid overlooking a primary psychiatric diagnosis. In this particular patient, the diagnostic work-up revealed high titers of positive VDRL-RPR and TPHA test results in both serum and cerebrospinal fluid, indicating a diagnosis of neurosyphilis. The history of substance abuse in this patient serves as a warning sign, as chronic exposure to these drugs can also lead to adverse effects, including psychosis, seizures, and cerebrovascular accidents, especially in individuals with preexisting risk factors. For the management of psychotic symptoms, risperidone was initiated, and infectious disease specialists administered procaine penicillin treatment to address the neurosyphilis.

This case report underscores the importance of interdisciplinary communication among various specialties, particularly primary care physicians, psychiatrists, neurologists, and infectious disease specialists.

#### 8.2.3. Research

Syphilis is a serious infectious disease, which can damage multiple organ systems and present with various clinical presentations [109,110,111]. In 4–9% of patients infected with syphilis (Traponema pallidum) and left untreated, neurosyphilis (NS) might develop as the consequence of involvement of the central nervous system [112,113]. The disease can either be asymptomatic or might present with multiple neurological and psychiatric symptoms such as seizures, ocular disorders, psychiatric/behavioral changes, and less likely tabes dorsalis or general paresis [114]. Neuropsychiatric symptoms can mimic any other primary psychiatric disorder and the patient might present with personality disorders, psychosis, delirium or dementia therefore initially refer to psychiatry clinics. The most challenging decision for psychiatrists dealing with these patients is to decide which patients should be screened for syphilis [109,110]. Sexually active young adults, individuals with multiple sexual partners or with a previous syphilis history, and drug/substance abuse history are some of the most frequent risk factors for syphilis infection and might require a routine screening [115]. Non-specific treponemal tests (RPR and VDRL) are the initial tests for screening and can be further confirmed by specific tests if they are positive [116]. Early diagnosis is extremely essential in NS, which is a medically curable disease if treated early, in order to prevent devastating outcomes [110]. A high index of suspicion followed by a full diagnostic workup (including serologic testing, lumbar punction, and neuroimaging), is the key to early diagnosis. A multidisciplinary approach is required to successfully identify and treat the neuropsychiatric manifestations of all infectious diseases [110].

#### 8.2.4. Agreement

The perspective aligns with the complexity of diagnosing neuropsychiatric symptoms and emphasizes thorough assessments, including history-taking, physical exams, neuroimaging, and laboratory tests, to identify underlying causes.

#### 8.2.5. Disagreement

While the analysis suggests that individuals with multiple sexual partners and a history of syphilis are at risk, it is important to also consider other factors like unprotected sex, sexual practices, and socio-economic factors that could contribute to syphilis transmission.

#### 8.2.6. Reflection

This examination prompts reflection on the complexities of differential diagnosis, especially in cases where organic and psychiatric causes overlap. It underscores the need for a cautious, multi-faceted approach to avoid misdiagnosis.

#### 8.2.7. Education (Key Messages)

- Neuropsychiatric symptoms can mimic any other primary psychiatric disorder, so it is important to consider the possibility of an underlying organic cause in patients presenting with these symptoms;
- Early diagnosis is essential in neurosyphilis, as it is a medically curable disease if treated early;
- A multidisciplinary approach is required to successfully identify and treat the neuropsychiatric manifestations of all infectious diseases;
- The risk of neurosyphilis is increased for sexually active young adults, individuals with multiple sexual partners, or those with a previous syphilis history;
- Early diagnosis and treatment of neurosyphilis is essential to prevent long-term central nervous system damage.

### 8.3. Psychiatric Point of View by E.E.B.

#### 8.3.1. Comprehension

This perspective highlights the complexity of diagnosing psychotic disorders, emphasizing the need for individual evaluation and consideration of organic causes, such as substance use and medical conditions.

#### 8.3.2. Appraisal

Psychotic disorders typically begin in the twenties and are characterized by impairment in functioning and social interaction. It is important to evaluate each patient individually, especially during the first episode, to rule out other medical conditions that may cause psychotic symptoms. Psychoses can be classified into three main categories: idiopathic psychoses, which have no identifiable cause; psychoses resulting from medical conditions, including neurodegenerative disorders; and toxic psychoses, which are caused by substances of abuse, prescribed medications, or toxins.

In order to exclude all these conditions, laboratory tests, neuroimaging, and EEG should be planned. The patient’s history of substance use in the past suggests a possible association between the psychotic symptoms and substance abuse. However, according to DSM-5 criteria, for a diagnosis of substance-induced psychotic disorder to be made, the symptoms must emerge within the first month following substance use. In this case, the patient’s last substance use was reported 8 months ago.

It Is important to note that substance use is observed in nearly half of individuals with psychotic disorders, and even if they cease substance use, they may still receive a diagnosis of psychosis during clinical follow-up. According to Aggarwal et al. (2012), during an average follow-up period of 6 months, 20.3% of patients initially diagnosed with substance-induced psychotic disorder experienced a change in diagnosis to either schizophrenia or affective psychosis.

The patient’s diagnosis has been confirmed through tests and clarified as neurosyphilis. Although there is no clear information regarding the date of syphilis transmission in the medical history, it can be estimated that it occurred approximately 2 years ago, during a period when the patient was unaware. Syphilis has stages including primary, secondary, latent, and tertiary. Following primary infection, the treponeme infiltrates the nervous system within a few days, and subsequent neurosyphilis can be classified into asymptomatic or symptomatic forms, as well as early (occurring 1 to 2 years after primary infection) or late stages.

Syphilis has been referred to as the “great imitator” due to its ability to produce a wide range of symptoms. Therefore, it should not be forgotten that the symptoms of neurosyphilis can manifest at any time after the infection, not necessarily following the stages of primary, secondary, and tertiary syphilis.

In terms of treatment, guidelines recommend parenteral penicillin G as the fundamental treatment for all stages of syphilis, as it has been proven effective for a long time and serves as the primary therapy for neurosyphilis as well. Additionally, the co-occurrence of other sexually transmitted diseases and syphilis is common. In the patient, other serological markers were evaluated both in blood tests and in the CSF, and they were found to be negative. This presented case report once again demonstrates the importance of considering differential diagnosis of other medical conditions before establishing a psychiatric diagnosis.

#### 8.3.3. Research

Syphilis is an infectious disease caused by the spirochete bacteria Treponema pallidum subspecies pallidum (T. pallidum). Research conducted on rabbits has demonstrated that treponemes can be detected in the CSF within hours of the initial infection [110]. Neurosyphilis exists in five forms: asymptomatic neurosyphilis, meningeal, meningovascular, general paresis, and tabes dorsalis [110,111]. With the widespread use of penicillin, the occurrence of late manifestations of neurosyphilis, such as tabes dorsalis and general paresis, has become less common.

The diagnosis of neurosyphilis can be challenging, as there is no standardized testing available. Instead, it is typically based on a combination of clinical evaluation and analysis of CSF findings. CSF VDRL, CSF treponemal tests, CSF pleocytosis, and CSF protein levels should be evaluated [113].

Penicillin G is considered the preferred treatment for neurosyphilis, although ceftriaxone may be a viable alternative therapy. The prognosis of patients with neurosyphilis primarily relies on the type of neurosyphilis and early detection of the disease. Patients who receive treatment years after the initial infection have a poorer prognosis and may experience persistent symptoms despite treatment [117]. Syphilis is also a diagnosis that requires being alert to other sexually transmitted diseases. HIV infection is the most common comorbidity among these infections. Untreated syphilis can increase the risk of transmission and acquisition of HIV infection. Additionally, patients with co-infection of HIV and syphilis may have an elevated risk of treatment failure, and their genital ulcers typically require a longer healing time compared to individuals with syphilis alone. This prolonged healing process can potentially increase the vulnerability to other sexually transmitted diseases [118].

Therefore, neurosyphilis should be included in the differential diagnosis of psychosis, and prompt treatment should be initiated.

#### 8.3.4. Agreement

The perspective agrees with the importance of considering substance-induced psychotic disorder, especially when the patient has a history of substance abuse. It notes the timeframe for diagnosing substance-induced psychosis and how it can persist beyond substance use.

#### 8.3.5. Disagreement

While the analysis presented the DSM-5 criterion of symptoms emerging within the first month of substance use for substance-induced psychotic disorder, there could be debates on the exact timeframe depending on individual cases and substances used.

#### 8.3.6. Reflection

The analysis delved into the intricate relationship between neurosyphilis and psychiatric symptoms, emphasizing its diverse clinical manifestations and the importance of early diagnosis and treatment.

#### 8.3.7. Education (Key Messages)

- Psychotic symptoms require a thorough evaluation to rule out organic causes, including substance use and medical conditions;
- Differential diagnosis is crucial in first-episode psychosis to consider various potential causes;
- Neurosyphilis can manifest as diverse psychiatric symptoms and should be considered in the differential diagnosis of psychosis;
- Prompt diagnosis and treatment of neurosyphilis are vital for optimal outcomes;
- Being attentive to the possibility of diagnostic changes over time enhances diagnostic accuracy.

### 8.4. Family Physician Point of View by N.T.

#### 8.4.1. Comprehension

This perspective highlights the importance of a comprehensive psychiatric evaluation and a multi-disciplinary approach in addressing a patient’s complex presentation involving psychiatric symptoms, substance abuse history, and positive serologic tests for syphilis.

#### 8.4.2. Appraisal

In this case, it is important for the family physician to consider several factors and make appropriate suggestions. First, given the patient’s significant psychiatric symptoms, including auditory hallucinations, paranoid delusions, and derealization, a comprehensive psychiatric evaluation should be conducted to assess the severity and nature of the symptoms. Additionally, the patient’s history of substance abuse and recent suicidal attempt highlight the need for a thorough assessment of her mental health, including screening for any co-occurring substance use disorders and assessing suicide risk. It is also crucial to address the patient’s previous history of anxiety symptoms and substance abuse, as they may contribute to her current presentation. Furthermore, the positive results on serologic tests for syphilis require immediate attention, and consultation with the infectious diseases department is warranted to initiate appropriate treatment and further evaluation. Finally, coordination with the infectious diseases service is necessary to ensure the patient receives the appropriate treatment for syphilis. The family physician should closely monitor the patient’s progress and coordinate with the multidisciplinary team to provide comprehensive care addressing both the psychiatric and infectious disease aspects of her condition.

#### 8.4.3. Research

Untreated psychosis is associated with poor quality of life and poor outcomes at first contact with mental health services. Help-seeking experiences and the roles of social networks should be investigated in the onset of psychosis. There are obstacles to the initial diagnosis of psychosis, including:

- Not attributing symptoms to psychosis [119];
- Distress about stigma [108];
- Concerns about seeking help from healthcare providers [120].

A study conducted in England found that communication problems existed between physicians working in primary care and patients with psychosis, especially those who presented during adolescence [118]. It has been revealed that only a small proportion of young people with psychosis seek help from a family doctor or other health institution at the time of onset. There is a need to directly involve the families of individuals with psychosis in the help-seeking process [119]. In the early forms of neurosyphilis, the cerebrospinal fluid (CSF), meninges, and vascular system are affected. In the late forms, the brain and spinal cord parenchyma are primarily affected. Patients may experience mental and cognitive changes that progress to psychosis and dementia. If left untreated, severe neurological and paralytic symptoms may develop and lead to death [108].

If neuropsychiatric symptoms are present in patients with unexplained systemic signs and symptoms, underlying medical conditions should be highly suspected [120]. Neuropsychiatric manifestations can be present in many medical conditions, such as infectious, autoimmune, endocrinological, metabolic, and neoplastic diseases. Detailed history and physical examination, laboratory tests, and imaging may be needed to determine whether a medical condition is the underlying cause of the neuropsychiatric symptoms [120,121].

#### 8.4.4. Agreement

The perspective aligns with the need for coordination between the family physician and infectious diseases department to ensure appropriate treatment for syphilis, while also highlighting the significance of monitoring the patient’s progress and providing comprehensive care.

#### 8.4.5. Disagreement

While the analysis discussed communication problems between physicians and patients with psychosis, there might be varying opinions on the extent and nature of these challenges and the best strategies to overcome them.

#### 8.4.6. Reflection

The perspective prompted reflection on the barriers that individuals with psychosis might face when seeking help and how involving families and addressing stigma can improve early intervention.

#### 8.4.7. Education (Key Messages)

- Psychiatric evaluation should assess the severity and nature of symptoms, considering co-occurring substance use disorders and suicide risk;
- A patient’s history, including anxiety symptoms and substance abuse, should be evaluated as potential contributors to their current presentation;
- Coordination with relevant specialists, such as the infectious diseases department, is crucial for appropriate treatment, especially when positive serologic tests are involved;
- A multi-disciplinary approach involving coordination with specialists ensures comprehensive care for patients with complex conditions;
- Early diagnosis and intervention are crucial for both psychiatric and underlying medical conditions presenting with neuropsychiatric symptoms.

## 9. Conclusions

- The case of chronic migraine with medication overuse underscores the significance of a multidisciplinary approach in managing complex neuropsychiatric conditions. Healthcare providers should prioritize patient awareness on the risks of medication overuse and the importance of adhering to appropriate guidelines. Treatment strategies should include a combination of prophylactic medications, nonpharmacological approaches, such as lifestyle modifications and trigger avoidance, and interventions like nerve blockades. Integrated management approaches, considering biological, psychological, and social factors, are essential for improving patient outcomes and enhancing their overall quality of life.
- This case highlights the importance of considering memory dysfunction with language and behavioral problems as potential indicators of an underlying neurological condition. Collaborative efforts between healthcare providers, such as family physicians, neurologists, and psychiatrists, are crucial for accurate diagnosis and effective management of these complex cases. A comprehensive evaluation including neurocognitive assessment, neuroimaging, and consideration of potential underlying causes is necessary to guide treatment strategies and improve patient outcomes.
- The case of refractory epileptic seizures with subjective sensory symptoms emphasizes the need for a collaborative approach involving family physicians, neurologists, and psychiatrists in managing complex neuropsychiatric cases. Comprehensive evaluation, including detailed patient history, neurological examination, neuroimaging, and EEG monitoring, is crucial for accurate diagnosis and treatment planning. The use of antiepileptic medications, tailored to the individual patient’s needs, along with adjunctive therapies like cognitive behavioral therapy, can help in achieving seizure control and improving the patient’s overall quality of life. Regular follow-up and ongoing collaboration between healthcare providers are essential for monitoring treatment response and optimizing management strategies.
- The case of bipolar affective disorder with normal pressure hydrocephalus highlights the importance of considering potential underlying medical conditions in patients with psychiatric symptoms. Collaborative efforts between family physicians, neurologists, and psychiatrists are crucial for accurate diagnosis and effective management. In this case, proper assessment for normal pressure hydrocephalus, including neuroimaging and cerebrospinal fluid analysis, is necessary. Treatment strategies may involve a combination of mood stabilizers for bipolar affective disorder and surgical intervention, such as cerebrospinal fluid diversion, for normal pressure hydrocephalus. Ongoing monitoring and interdisciplinary collaboration are essential for optimizing treatment outcomes and improving the patient’s overall well-being.
- The case of postherpetic neuralgia in a patient with bipolar affective disorder highlights the need for a comprehensive and integrated approach to managing complex neuropsychiatric cases. Collaboration between healthcare providers, including family physicians, neurologists, and psychiatrists, is essential for accurate diagnosis and effective management. Treatment strategies may involve a combination of antiviral medications for the herpes zoster infection, analgesics for pain management, mood stabilizers for bipolar affective disorder, and interventions such as nerve blocks or neurostimulation for neuropathic pain. Close monitoring, patient education (key messages), and ongoing interdisciplinary communication are necessary to optimize treatment outcomes and enhance the patient’s quality of life.
- The case of psychosis with recurrent attacks and substance abuse highlights the complex nature of neuropsychiatric conditions and the importance of a collaborative approach in their management. Healthcare providers, including family physicians, neurologists, and psychiatrists, need to work together to assess the underlying causes of psychosis, address substance abuse issues, and develop a comprehensive treatment plan. This may involve a combination of pharmacotherapy for psychosis, substance abuse counseling and rehabilitation programs, and supportive therapies such as cognitive behavioral therapy. Close monitoring, ongoing communication, and a holistic approach that considers both the biological and psychosocial aspects of the patient’s condition are crucial for achieving successful outcomes and promoting long-term recovery.

**Figure 1:**
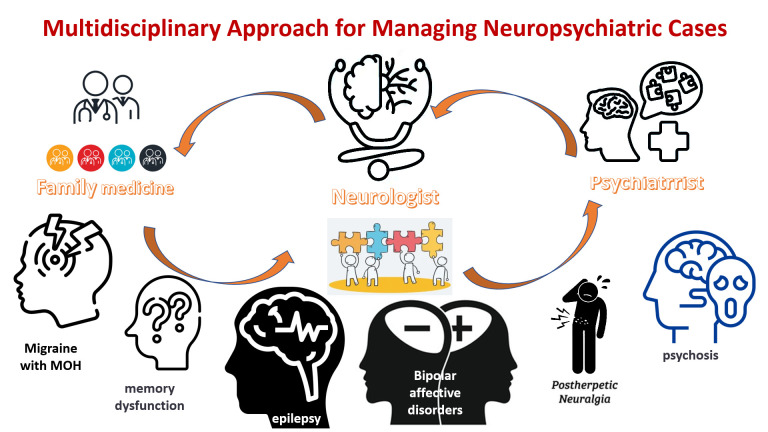
Multidimensional approach summarized model to same cases with known recurrent neuropsychiatric disorders.

In conclusion, the multidisciplinary case-based discussions on migraine, dementia, epilepsy, mood disorders, neuralgia, and psychosis underscore the importance of collaborative efforts between family physicians, neurologists, and psychiatrists in managing complex neuropsychiatric cases (see Figure 1). A biopsychosocial approach that considers biological, psychological, and social factors is essential for accurate diagnosis, comprehensive treatment planning, and improving patient outcomes. By promoting interdisciplinary collaboration, patient education, and integrated management strategies, healthcare providers can ensure that patients with these multifaceted conditions receive the best possible care and support for their overall well-being.

## 10. Takeaway Messages

- Neuropsychiatric cases necessitate a multidisciplinary approach involving family physicians, neurologists, and psychiatrists for effective management;
- The biopsychosocial model is crucial in understanding and addressing the complex nature of neuropsychiatric conditions, considering biological, psychological, and social factors;
- Collaboration between healthcare providers ensures comprehensive and tailored care for patients with complex and multifaceted needs;
- The CARE framework (Case Report, Appraisal, Research, and Education) guides reporting and evaluation of case reports, promoting comprehensive and effective care;
- Patient education on medication overuse, triggers, and treatment options plays a pivotal role in the management of conditions such as chronic migraines;
- Psychological comorbidities, including anxiety and depression, are prevalent in individuals with migraines, highlighting the importance of referral to psychiatry and psychological treatment options;
- Longitudinal and holistic approaches, supported by patient headache diaries, aid in accurate diagnosis and treatment planning for headache cases;
- Collaboration between healthcare providers and patient empowerment through therapeutic patient education enhance disease management and improve outcomes;
- Comorbidities and underlying medical conditions, such as normal pressure hydrocephalus, should be considered in patients with psychiatric symptoms for accurate diagnosis and tailored treatment plans;
- Integrated approaches combining pharmacotherapy, psychotherapy, and surgical interventions may be necessary for managing complex neuropsychiatric cases effectively.

## Data Availability

Not applicable.
